# Volatile Organic Compounds (VOCs) of Endophytic Fungi Growing on Extracts of the Host, Horseradish (*Armoracia rusticana*)

**DOI:** 10.3390/metabo10110451

**Published:** 2020-11-08

**Authors:** Tamás Plaszkó, Zsolt Szűcs, Zoltán Kállai, Hajnalka Csoma, Gábor Vasas, Sándor Gonda

**Affiliations:** 1 Department of Botany, Division of Pharmacognosy, University of Debrecen, Egyetem tér 1, 4032 Debrecen, Hungary; plaszko.tamas@tutamail.com (T.P.); szucs.zsolt@science.unideb.hu (Z.S.); vasas.gabor@science.unideb.hu (G.V.); 2 Doctoral School of Pharmaceutical Sciences, University of Debrecen, 4032 Debrecen, Hungary; 3 Department of Genetics and Applied Microbiology, University of Debrecen, 4032 Debrecen, Hungary; kallai.zoltan@tarcalkutato.hu (Z.K.); hajnalka.csoma@outlook.com (H.C.); 4 Research Institute for Viticulture and Oenology, 3915 Tarcal, Hungary

**Keywords:** VOC, glucosinolates, endophytic fungi, thioglucosidase, nitrile

## Abstract

The interaction between plant defensive metabolites and different plant-associated fungal species is of high interest to many disciplines. Volatile organic compounds (VOCs) are natural products that are easily evaporated under ambient conditions. They play a very important role in inter-species communication of microbes and their hosts. In this study, the VOCs produced by 43 different fungal isolates of endophytic and soil fungi during growth on horseradish root (*Armoracia rusticana*) extract or malt extract agar were examined, by using headspace-gas chromatography-mass spectrometry (headspace-GC-MS) and a high relative surface agar film as a medium. The proposed technique enabled sensitive detection of several typical VOCs (acetone, methyl acetate, methyl formate, ethyl acetate, methyl butanol isomers, styrene, beta-phellandrene), along with glucosinolate decomposition products, including allyl cyanide and allyl isothiocyanate and other sulfur-containing compounds—carbon disulfide, dimethyl sulfide. The VOC patterns of fungi belonging to *Setophoma*, *Paraphoma*, *Plectosphaerella*, *Pyrenochaeta*, *Volutella*, *Cadophora*, *Notophoma,* and *Curvularia* genera were described for the first time. The VOC pattern was significantly different among the isolates. The pattern was indicative of putative myrosinase activity for many tested isolates. On the other hand, endophytes and soil fungi as groups could not be separated by VOC pattern or intensity.

## 1. Introduction

Volatile organic compounds (VOCs) are natural products that are volatile under ambient conditions. They are mostly produced by plants, bacteria, or fungi. Examination in recent years has revealed a surprising diversity of compounds, with very interesting biological roles. Chemically, the compounds include acids, alcohols, aldehydes, esters as well as terpenoids. They are usually analyzed with special sampling methods, as reviewed in [1].

The VOC profile can also be used in chemotaxonomic profiling. Examples include solid phase microextraction gas chromatographic (SPME-GC) profiling of *Fusarium verticilloides, F. oxysporum, F. poae*, and *F. equiseti* with various identified sesquiterpenes, including ones indicative of toxin production [2]. The strains also produce short chain alcohols, their esters, ketones, and hydrocarbons.

Fungal VOCs are very important in mediating interactions of fungi with other organisms *in situ*. Inter-species effects include involvement in formation and regulation of symbiotic associations, the distribution of fungi with various lifestyles via antifungal activities, phytotoxicity, and insect attractant or repellent activities [3]. Self-regulatory function was described for 1-octen-3-ol, produced by various fungi, which acts as a self-inhibitor of spore germination in *Penicillium paneum* [4], *Aspergillus nidulans* [5], and *Lecanicillium fungicola* [6].

Of high interest are endophytic and other isolates of *Muscodor sp.* (mostly *M. albus*), which can be readily used in agricultural applications. In particular, the fungal VOC mixture inhibits the growth of many plant pathogenic fungi on fruits [7]. A *M. yucatanensis* isolate was shown to produce a mixture of hydrocarbons, esters, as well as sesquiterpenes [8]. VOCs produced by *Trichoderma viride* [9], a biocontrol strain of *Cladosporium cladosporioides*, enhanced plant growth in in vitro models [10].

A significant plant chemical defense system also produces volatile natural products. The *Brassica* vegetables produce glucosinolates (GLs, glucosides of thiohydroximates), which are deglycosylated by myrosinase and subsequently, rearrange to isothiocyanates or other decomposition products upon tissue damage [11]. Depending on the side chain, these products can be very volatile, as in the case of the widespread GLs sinigrin (allyl glucosinolate) and gluconasturtiin (phenylethyl glucosinolate). While glucosinolates themselves do not inhibit fungal growth [12], the strong in vitro antifungal activity of the isothiocyanates (ITCs) has been recognized as early as the 1960s [13].

VOCs are also known to play a role in plant–microbe interactions. However, during a plant’s defense against a fungal pathogen in vivo, the situation is more complex than it would seem at first glance. It was shown that the *tgg1/2* mutant *A. thaliana* (no myrosinase, nor ITCs) was more sensitive to *A. brassicicola* and *B. cinerea* B05.10, but not a grape *B. cinerea* isolate, suggesting a role of ITCs in fungal defense [14]. On the other hand, a specific myrosinase called PEN2 is also involved in defense, and aids the conversion of indole glucosinolates to other types of products, including raphanusamic acid and indoyl-methyl amine—possibly via ITCs as intermediates [15]. Additionally, indolic glucosinolates (decomposed to non-volatile products) seem to be even more important in restricting fungal invasion, as shown in *A. thaliana* knock-out models: the natural root endophyte *Colletotrichum tofieldiae* becomes pathogenic in lines without the ability to biosynthesize indolic glucosinolates [16]. A similar increase in susceptibility was shown for *Plectosphaerella cucumerina* [17].

Several fungi have the ability to decompose or utilize glucosinolates as a carbon source. The latter seems to be more widespread among endophytes than in a random set of soil fungi [18]. The type and fate of decomposition products have always been a major question regarding this context, as dealing with fungitoxic isothiocyanates requires much biochemical effort from the fungal consumer of the glucosinolates [19]. The corresponding nitriles and epithionitriles are generally accepted to be less toxic to plant pathogens and herbivores than the default decomposition products, the ITCs [20].

The exposure of fungi to various ITCs result in a transcriptional response showing overexpression of oxidative stress defense genes in the Brassicaceae pathogen *Alternaria brassicicola* [21]. Induced genes include glutathione S-transferases, γ-glutamylcysteine synthetases, thioredoxins, oxidoreductases, and heat-shock proteins. Upregulation of antioxidant enzyme activities upon horseradish essential oil (composed mainly of ITCs) exposure was also observed in *C. albicans* [19].

In some cases, ITC was shown to be the main fungal decomposition product of glucosinolates [22], while other fungi rather produced nitriles [23,24]. In other instances, it is unclear what decomposition products were produced [24,25]. It is possible that allyl isothiocyanate (AITC) was actually the main product, but as it conjugates with glutathione (GSH) very rapidly, it was not detectable by GC-MS; only non-volatile conjugates could be found [18].

There are several studies published on VOCs from Brassicaceae vegetables, but these exclusively use the plant myrosinase as the decomposing agent. Usually, the headspace-SPME approach is used [26,27], with some sort of GC-MS detection. The major disadvantages of SPME fibers are ageing of the fiber material, selectivity of the adsorption of volatiles onto the fiber, and possible saturation effects resulting in carry-over. Despite these, this technique is widely used for the screening of volatiles due to its sensitivity, but it is rather used in a semi-quantitative fashion, also reflected in the studies [26,27]. For some analytes, however, much higher sensitivity can be obtained using a proper fiber than by injecting headspace air. Semi-quantitative data are sometimes reinforced by usage of internal standards, typically octanol [28,29].

The aim of the current study is to examine the VOC production of diverse endophytic fungi when grown on the extract of their host plant, *Armoracia rusticana* (horseradish), with special respect to possible sulfur-containing glucosinolate decomposition products. The very high amounts of glucosinolates [30] in horseradish make it a suitable candidate for such studies.

An additional hypothesis was that endophytic fungi and non-host-derived fungi decompose the plant extract in a slightly different manner.

## 2. Results

### 2.1. Identified Fungi

The list of identified fungi includes endophytic fungi from horseradish and soil fungi from the same soil (Table 1). E1–E7 isolates were identified in our recent study [18]. The gene sequences of other isolates were matched against entries of the National Center for Biotechnology Information Nucleotide collection via BLAST (Basic Local Alignment Search Tool) [31]. Identification was carried out by sequencing one of the internal transcribed spacer (ITS) region, α-actin, or calmodulin genes. When a single gene sequence did not result in species-level resolution, only the genus of the isolate is listed. The gene sequences used for identification are listed in Supplementary Material 1.

### 2.2. Identified VOCs

The set of fungi emitted VOCs belonging to various chemical compound classes, including esters, short chain alcohols, a short chain acid, an aromatic compound, a monoterpene, as well as several sulfur-containing VOCs and nitriles, possibly originating from the glucosinolates of the horseradish extract medium. The spectrum and retention time of several compounds were compared to those of authentic standards; the rest were putatively identified by the National Institute of Standards and Technology (NIST, Gaithersburg, MD, USA; Version 2.0 g, build 19 May 2011) library search. The list of (putatively) identified compounds was summarized in Table 2.

**Figure 1:**
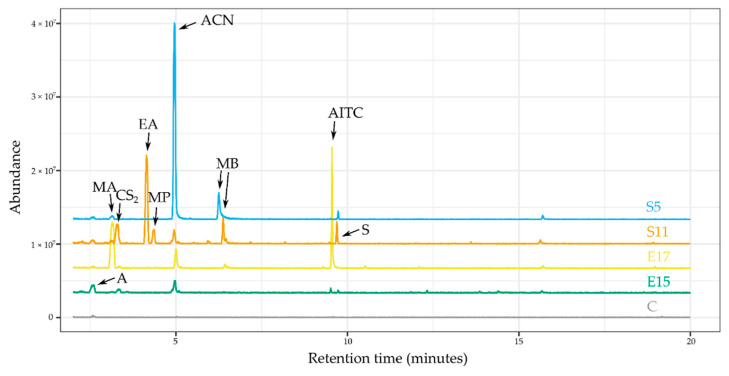
Representative total ion chromatograms (TICs) from the headspace of horseradish extract incubated with various endophytic fungi and soil fungi. Chromatogram traces (from bottom to top): C—control, non-inoculated horseradish extract; E15—*Phomopsis sp.*; E17—*Colletotrichum sp.*; S11—*Fusarium sp.*; S5—*Curvularia sp.* Abbreviations: A—acetone; ACN—allyl cyanide; AITC—allyl isothiocyanate; carbon disulfide—CS_2_; EA—ethyl acetate; MA—methyl acetate; MB—methyl-1-butanol (two isomers); MP—methyl-1-propanol; S—styrene.

The identified compounds include several compounds that unambiguously come from glucosinolate decomposition: allyl cyanide, allyl isothiocyanate, phenylpropionitrile, and 2-phenylethyl isothiocyanate; and two sulfur-containing compounds of likely glucosinolate origin: carbon disulfide and dimethyl sulfide. Altogether, 6 esters were detected: ethyl acetate, ethyl propionate, methyl acetate, methyl-1-butanol acetate, methyl formate, and propyl acetate; and two short chain alcohols were also identified: two methyl-1-butanol isomers and methyl-1-propanol. Other compound classes include two aromatic compounds (benzaldehyde, styrene), an organic acid (acetic acid), a ketone (acetone), and a monoterpene (beta-phellandrene). There were 18 peaks that could not be identified, mostly due to low signal-to-noise ratios, resulting in poor library match scores. A few representative chromatograms of different fungi, with some of the identified peaks marked, are shown in Figure 1.

The proposed procedure was shown to be very suitable to detect allyl cyanide (ACN) production from glucosinolates. In our recent study [18], despite efforts, ACN, contrary to AITC, could not be detected by adsorption on charcoal, while in the current study, the growth of several fungi resulted in ACN emission. It is important to state that none of the sulfur-containing glucosinolate decomposition compounds were detectable from fungi growing on malt extract agar (MEA) medium. Considerable amounts of the other identified compounds were detected in the following strains: acetone—S2, S24; acetic acid—S10, S25; methyl-1-butanol—E1, E3, E8, E9, E12, E17, S1, S3, S10, S12, S25; methyl-1-propanol—E1, E3, E8, E9, E12, S3, S10, S12; beta-phellandrene—E15; styrene—S11; benzaldehyde—E7.

### 2.3. Performance of the GC-MS Method and the Proposed Cultivation Technique

The proposed procedure for evaluation of VOCs from endophytic fungal culture is a high relative surface agar film created by dispersing cooling agar medium by rotation, so that it covers the bottom and the lower side of the headspace vial. The compound pattern and peak intensities of 7 fungi cultivated using the proposed strategy and the reference strategies (liquid, low relative surface agar) were compared in a pilot (see Supplementary Table S2 for raw data). Twenty-two fungus–metabolite pairs were most abundant with the proposed method, compared to 27 and 12 in the case of liquid and simple agar media (in 65 cases, the compound–fungus pair was not detected in any of the treatments). When comparing the abundances of compounds from samples using the proposed technique with those grown on liquid medium, a fold-change of 0.7219 was observed. The cultivation technique advantage was highly fungus-specific; the fold-change of TIC vs. that measured in liquid media ranged from 0.016 to 5.708. Of more interest, it was shown that the proposed technique gives the highest amount of GL-derived VOCs: AITC, CS_2_, and ACN showed the highest abundance with the proposed technique in the case of 4 of 7 producers, 5 of 5 producers, and 4 of 6 producers, respectively. However, in the case of other metabolites, the liquid medium was typically the best choice: the overall peak abundance (TIC) was the highest on liquid cultures in 4 of 7 cases (with the proposed method being the best in 3 fungi). As the focus of the current study was potential glucosinolate decomposition products, the whole study was run using the proposed technique.

The GC-MS method performance was checked by injection of small amounts of all authentic standard mixtures (synthetic quality control (QC) samples) along the analysis, and calculation of the standard deviation along the chromatographic run. This has shown that more volatile compounds (including ACN, AITC, and all short chain esters and alcohols) show good to excellent reproducibility: median intraday relative standard deviation values for AITC, ACN, Me_2_S, and CS_2_ were 0.076, 0.082, 0.123, and 0.137 in QC samples, respectively. On the other hand, less volatile compounds (including PEITC and PCN) were less reliably quantified and carry-over phenomena were also observed. Of importance is the low LOQ for the analytes of interest, which ranged from 2.85 × 10^−7^ to 8.84 × 10^−6^ mmol (PEITC and Me_2_S, respectively). Additional metrics on method performance can be found in Supplementary Table S3.

In the case of these compounds, liquid–liquid extraction would have been more accurate for quantitative determination and had to be omitted from later analysis.

### 2.4. Differences among VOC Patterns of Fungi

**Figure 2:**
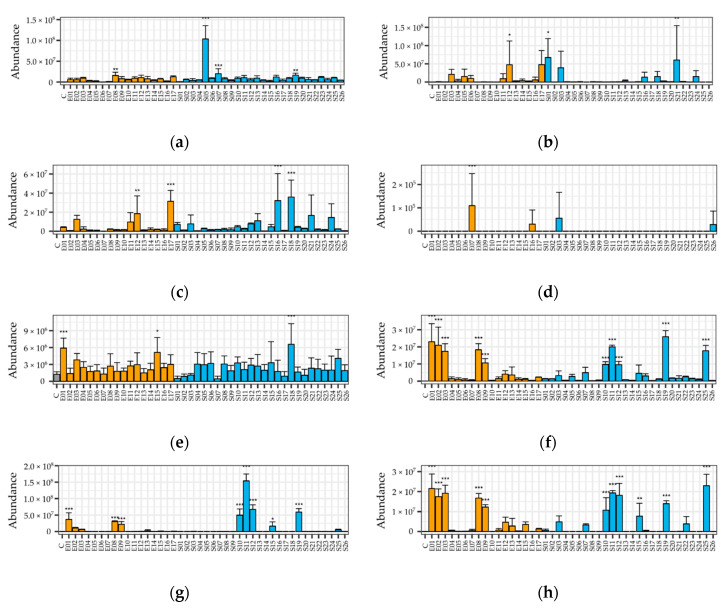
Abundance of selected VOC compounds in the headspace of horseradish extract medium, fermented by endophytic and soil fungi. Presented data are average ± sd of 4 replicates, day 3 and 4 of 2 biological replicates per treatment. The *x* axis shows the id of the fungus; respective species names can be found in Table 1. Color coding refers to the fungal isolate type: grey—control; orange—endophytic fungus from horseradish roots; blue—soil fungi. Subplots: (**a**) Allyl cyanide (ACN)*; (**b**) Allyl isothiocyanate (AITC)*; (**c**) Carbon disulfide (CS_2_)*; (**d**) Dimethyl sulfide* (Me_2_S); (**e**) acetone*; (**f**) 3-methyl-butanol isomer; (**g**) Ethyl acetate*; (**h**) Possibly sulfur containing, unidentified compound. Compounds marked with an asterisk were identified by comparison with authentic standards. Bars marked with asterisks denote statistically significant differences vs. control (ANOVA, followed by Dunnett post hoc test): *, *p* < 0.05; **, *p* < 0.01; ***, *p* < 0.001.

Several fungi emitted short chain alcohols like methyl-1-butanol isomers, acetone, and esters such as methyl formate or ethyl acetate, as well as possible glucosinolate breakdown products also being detected (Supplementary Table S1). The presence of these compounds in trace amounts in the headspace of the non-inoculated horseradish extract control suggests that they could be emitted by a spontaneous decomposition reaction. However, a notable number of endophytic and soil fungi emitted significantly higher amounts of these compounds, compared to the control samples (Figure 1 and Figure 2a,b). The most prominent strains, which emitted a significant amount of acetone, were E1—*Fusarium oxysporum*, E15—*Phomopsis sp.*, and S18—*Penicillium sp.* Acetone as a VOC is a common substance from fungi, described from endophytes like *M. albus* [32]. Incubation with almost every strain increased the production of ACN in the headspace, giving significant differences in the case of E8, S7, S19 (all *Fusarium sp.*), and S5, *Curvularia* (Figure 2a). Interestingly, other *Fusarium* strains did not emit a considerable amount of this compound. AITC was present in much fewer headspace samples (Figure 2b), compared to the other glucosinolate decomposition products and it was significant in 3 fungi: E12—*Plectosphaerella sp*., S1—*Notophoma sp*., and S21—*Paraphoma sp*.

The following compounds were not detected in the horseradish extract control samples, hence, they are likely produced by the fungi themselves: methyl-1-butanol acetate, methyl-1-propanol, methyl-1-butanol, acetic acid, carbon disulfide, dimethyl sulfide, ethyl acetate, methyl acetate, methyl formate, ethyl propionate, propyl acetate, benzaldehyde, beta-phellandrene, styrene, and 18 unidentified compounds. From the sulfuric compounds, carbon disulfide was identified in almost every fungi growing on horseradish extract, but four strains showed a remarkable emission i.e.,: E21 —*Plectosphaerella sp.*, E17—*Colletotrichum sp.*, S16—*Fusarium sp.*, and S18—*Penicillium sp.* (Figure 2c). Another VOC possibly containing sulfur was detected (*m/z* = 72, rt 3.33 min), which has a spectrum very similar to that of thiirane, yet the two compounds turned out to be different when comparing with the authentic standard. This compound was emitted in significantly high amounts by E12—*Plectosphaerella sp.*, E17—*Colletotrichum sp.*, S16—*Fusarium sp.*, and S18—*Penicillium sp.* (Figure 2h). Dimethyl sulfide was also detected; its presence was proven by the authentic standard. Interestingly, it was only emitted by 4 strains: E7—*Oidiodendron cerealis*, E16—*Cadophora sp.*, S3—*Curvularia sp.*, and S26—*Penicillium sp.* (Figure 2d); not even traces of this substance were found in any other samples.

A small group of endophytic and soil fungi (E1, E8, E9, S10, S11, S12, S15, S19, and S25) all belonging to the *Fusarium* genus emitted a significant amount of esters like ethyl acetate (Figure 2g) and methyl acetate (both identity verified with authentic standard) in a very distinctive pattern. Two other possible esters, ethyl propionate and propyl acetate, and short chain alcohols, methyl-1-propanol and methyl-1-butanol (Figure 2f, identified by NIST library), were emitted significantly by almost the same strains mentioned before. Methyl-1-butanol (or isoamyl alcohol) is a promising fuel additive [33] and the precursor of the important flavoring and fragrance compound isoamyl acetate [34]. E2—*Macrophomina phaseolina* and E3—*Fusarium oxysporum* were the only strains that emitted a significant amount of the alcohols, but insignificant amounts of esters.

Another ester, methyl formate, was emitted in significant amounts by S13—*Penicillium sp.*, S16—*Fusarium sp.*, and S18—*Penicillium sp.* and methyl-1-butanol acetate was emitted by E12—*Plectosphaerella sp.*, E17—*Colletotrichum sp.*, and S21—*Paraphoma sp*. In terms of abundancy, some less common compounds were: acetic acid and styrene were only emitted by S11—*Fusarium sp.* in a considerable amount, benzaldehyde by E2—*Macrophomina phaseolina*, S19—*Fusarium sp.*, and beta-phellandrene by E15—*Phomopsis sp*.

## 3. Discussion

### 3.1. Fungi

The isolated endophytic fungi include somewhat uncommon genera like *Volutella sp.*, but are rather dominated by common endophytic species of Brassicaceae plants. The overall pattern contains many genera that were previously described from *Arabidopsis thaliana*, also a member of Brassicaceae [35]. Examples include genera *Phoma*, *Plectosphaerella*, *Phomopsis*, and *Volutella*. The soil fungal set showed a much lower diversity; most species either belong to *Fusarium sp.* or *Penicillium sp.*, with a few additions of *Curvularia sp.* This is in concordance with the findings of [36].

### 3.2. Performance of Proposed Cultivation Technique

Our proposed method yielded diverse VOC profiles for most tested filamentous fungi. The closed headspace vial in the method likely results in microaerophilic conditions, which facilitates VOC production of endophytes [37].

In the case of several fungal genera, the VOC pattern was described for the first time, to our best knowledge, including the *Setophoma*, *Paraphoma*, *Plectosphaerella*, *Pyrenochaeta*, *Volutella*, *Cadophora*, *Notophoma*, and *Curvularia* genera. Detailed lists of VOCs were given in Supplementary Table S1. Some representatives of a few genera have been already VOC profiled before, like *Macrophomina* [38], *Oidiodendron* [39], *Phomopsis* [40], *Colletotrichum* [41], *Penicillium* [42], *Aspergillus* [43], and *Fusarium* [2]. Numerous compounds were identified in those studies as well, such as methyl-1-propanol, methyl-1-butanol, acetone [40], methyl-1-butanol acetate, benzaldehyde [41], and styrene [42].

The sulfur-containing and other VOCs produced by these fungi are likely to contribute to their competitiveness during conditions when the plant host material is available as a nutrient. These situations include feeding on exudates of the plant [44]—which is described to contain glucosinolates in *Brassica rapa* ssp. *Rapa* [45]. The conversion is likely to contribute to the changes induced by microbial composition by Brassicaceae plants in their rhizosphere [46].

### 3.3. Glucosinolate Decomposition by Fungi

The ability of fungi to decompose glucosinolates has been described long ago by [47] in *Aspergillus sydowi*. Since then, the phenomenon has been shown for many fungal strains, including but not limited to *A. niger* [48], *A. clavatus* and *Fusarium oxysporum* [24], *Aureobasidium pullulans*, *Fusarium venenatum*, *Trichoderma reesei* [49], as well as *Macrophomina phaseolina*, *Setophoma terrestris*, and *Phoma radicina* [18].

On the other hand, glucosinolate decomposition products are frequently problematic to detect. For example, a study [25] failed to detect usual glucosinolate hydrolysis products (isothiocyanates, nitriles, amines, or other products) during in vivo fermentation. In the case of allyl cyanide, the cause might have been poor enrichment via liquid–liquid extraction, while ITCs can be present in their thiol conjugate forms when produced intracellularly, as we recently concluded [18].

As the sulfur-containing compounds were totally absent in the headspace of fungi grown on MEA, the compounds are likely the results of glucosinolate decomposition. As the QC samples showed good Relative Standard Deviation (RSD) values for AITC, ACN, CS_2_, and Me_2_S, while PEITC and PCN reproducibility was compromised; we hereby deal with the more convincing former four only.

The presented method showed excellent detection capability for ACN from sinigrin: 4 of 45 strains showed significantly more amounts of this decomposition product, but almost all strains increased the amount of ACN in the headspace to some extent, compared to the control (Figure 2a). This does not have to be the result of a specific enzymatic activity; a shift in pH or ionic milieu might also contribute to accelerated spontaneous decomposition.

In contrast, much less strains produced AITC and only three species have shown significant AITC release. This product is unquestionably the decomposition product of sinigrin. As discussed in our recent study [18], a possible mechanism reducing the amount of vaporized AITC is intracellular decomposition of glucosinolates, which is followed promptly by AITC conjugation with a thiol of a protein or GSH, rendering detection by GC-MS impossible. This is not true for allyl nitrile, which is less likely to be trapped inside, being much less reactive and extremely volatile. Easy evaporation, relatively high polarity resulting in low liquid–liquid extraction enrichment, and low affinity of the allyl cyanide towards SPME materials might have also contributed to failed attempts to detect the compound in similar experiments. As the extracts were mostly the same, this might have been the case in our recent study as well [18].

An interesting observation is that many VOC mixtures contain carbon disulfide as a decomposition product; in 4 cases, the difference is significant. The CS_2_ release can be also be a result of non-enzymatic reaction of ITCs [50], as well as a detoxification pathway of ITCs by the fungi. This is well supported by the correlation between AITC and CS_2_ levels in the headspace (*R^2^* = 0.608), but this is not shown in case of dimethyl sulfide, which is detected in a few instances only (*R^2^* = 0.029). Dimethyl sulfide thus can be the result of an alternative decomposition/detoxification pathway.

### 3.4. Lack of VOC Pattern Significant Differences between Host Endophytes and Soil Fungi

The VOC pattern was highly indicative of the isolate, as several compounds showed highly significantly differences among different isolates, including ACN (*p* = 5.55 × 10^−48^), CS_2_ (*p* = 5.40 × 10^−13^) and AITC (*p* = 3.48 × 10^−4^).

However, the overall pattern and the individual compound abundances were not significantly different between the soil and the endophytic fungi as grouped sets. The taxonomic variability of the endophyte group (Table 1) and the lack thereof in the case of soil fungi might have been the reason of the inability to distinguish these groups in a statistically significant manner. The difference of the abundance of compounds between *Fusarium* strains and strains of other genera is quite prominent (Figure 1, Table 1). Despite this fact, even when comparing only the *Fusarium* strains of the two groups of fungi, no significant differences were found after correction for testing a high number of statistical hypotheses.

## 4. Materials and Methods

### 4.1. Chemicals

Reagents were at least of analytical purity. Media components (malt extract, peptone, sodium nitrate, potassium hydrogenphosphate, magnesium sulfate, potassium chloride, ferrous sulfate) were purchased from Reanal (Budapest, Hungary); glucose-monohydrate, streptomycin, chloramphenicol, and dichloran were from VWR (Radnor, PA, USA). Acetone, allyl isothiocyanate, allyl cyanide, 2-phenylethyl isothiocyanate, phenylpropionitrile, dimethyl sulfide, carbon disulfide, methanol, ethyl acetate, methyl acetate, and methyl formate were purchased from Sigma Aldrich (St. Louis, MO, USA). Pure sinigrin was obtained from Phytoplan (Heidelberg, Germany). Type I (18.2 MΩ cm^−1^) water purified by a Human Zeneer Power I water purification system (Human corporation, Seoul, Korea) was used throughout the study.

### 4.2. Standard Microbiological Media

Malt extract agar (MEA, 20 g L^−1^ malt extract, 20 g L^−1^ glucose monohydrate, peptone 1 g L^−1^), Saboraud glucose agar (SGA, 40 g L^−1^ glucose monohydrate, peptone 10 g L^−1^, pH 5.6 ± 0.2), or potato dextrose agar (PDA, potato infusion equivalent to 200 g L^−1^, 20 g L^−1^ glucose monohydrate, pH 5.6 ± 0.2) were used as media. Media were solidified with 2% agar.

### 4.3. Horseradish Extract Medium

The horseradish extract (HRE) medium was prepared as in our recent study [18], as follows: about 500–1000 g of healthy horseradish roots were cut into large pieces and subsequently cooked in water for 30 min to inactivate the myrosinase followed by homogenization with MeOH in a 3:2 solvent to fresh weight ratio. The mixture was subsequently boiled (80 °C) under reflux for 30 min. The resulting extract was filtered and evaporated to dryness in a rotary evaporator and resuspended in roughly the amount of water that was present in the roots (typically 70% of fresh weight). The liquid was filtered sterile on 0.20 μm PES membranes after prefiltrations, and stored at −24 °C before use. If necessary, the liquid was supplemented with 2% autoclaved agar. A single batch was used throughout the study, which contained 1856 µg mL^−1^ sinigrin, 239.8 µg mL^−1^ gluconasturtiin, 6.96 µg mL^−1^ glucoiberin, and 41.33 µg mL^−1^ glucobrassicin.

### 4.4. Isolation of Fungal Strains

Subsets of both fungal sets were used from our recent study [18]. In the current paper, 10 additional endophytes and 16 additional soil fungi were tested, meaning 17 endophytic and 26 soil fungal isolates in total.

Endophytic fungi were isolated from healthy horseradish (*Armoracia rusticana*) roots, collected from a cultivated population near Debrecen. Collection complied with local legislation and guidelines. Surface-sterilization was accomplished by 4-fold diluted commercial bleaching solution, followed by several rinses with sterile distilled water. The roots were thereafter cut into pieces (approximately 2 × 2 × 1 cm in size) and placed on standard media for endophyte isolation. Surface sterility was checked by imprinting or rinse fluid colony counting on the same type of medium. When no growth in these negative control plates was observed, the explant batch was considered free of surface contaminants. Incubation took place at room temperature in darkness. The fungi that appeared at the edge of the root pieces were considered endophytes. The isolated endophytic fungi are referred to as E1–E17 throughout the manuscript.

As a reference set, a set of soil fungi was also obtained from the Debrecen site, from a plant-free sampling point. About 1 g of soil was suspended vigorously in 10 mL of sterile water. This stock suspension was 10^3^-fold diluted and 100 μL of the diluted suspension was spread onto PDA or SGA plates, supplemented with 50 mg L^−1^ streptomycin and 50 mg L^−1^ chloramphenicol, and optionally, 2 mg L^−1^ dichlorane. The fungi that appeared were submitted to continuous subcultures to obtain pure strains. The soil fungi are referred to as S1–S26 throughout the paper.

### 4.5. Fungal Inoculum Preparation and Headspace GC-MS Measurements

For the initiation of experiments, liquid suspensions of the fungi were used to ensure uniform inoculation of samples. Fungi were grown in 30 mL of malt extract broth in shaken cultures at room temperature, 200 rpm, for 7–10 days. The fungal mycelia were disintegrated in a MiniMix CC lab blender (Interscience, Saint-Nom-la-Bretèche, France) using sterile BagPage (Interscience) plastic bags. After blending, the disintegration was facilitated manually by gently rolling a plastic rod on the bags. The suspension from the bags was centrifuged on 13,500 rpm for 2 min and the mycelia were washed with sterile water. The suspensions of the fungal inoculums were thereafter stored at 4 °C before the experiments. As the species differed in morphology and the ability to form conidia, dry weight per mL was chosen as a means of standardization. Dry weight was determined gravimetrically after lyophilization of the suspensions of the inoculum overnight after homogenization.

For the measurement of VOCs emitted by the different fungi strains, autoclaved headspace vials were used as the incubation vessels. Fungal emissions were tested on one of the following layouts: (a) The proposed method—1 mL of the warm, agar supplemented medium was pipetted into the vials which was rotated until the media solidified like a thin film on the bottom and adjacent lower portion of the vial walls; (b) 1 mL warm, agar supplemented medium was pipetted to the bottom of the vial and allowed to solidify; (c) 1 mL liquid medium was pipetted into the vial.

Thereafter, a 500 µL aliquot of fungal suspension, containing 500 µg dry weight equivalent fungal inoculums, was pipetted into the vials. After half a minute of gently rotating the vials, the excess water was removed and the vials were left open for 30 min in a laminar air flow box, so that the liquid residues could evaporate. After this step, the vials were closed and incubated at room temperature, for 3 days before the first measurement.

The headspace of each fungus was sampled on day 3 and 4 post inoculation; the experiment was run with two biological replicates, meaning 4 replicates altogether per fungus. Malt extract controls were used to qualitatively exclude the presence of sulfur-containing compounds; these were prepared in a single biological replicate and injected on day 3 and day 4 post inoculation.

### 4.6. Headspace Measurement Parameters

VOC profiling of the fungal headspace was carried out using a Bruker Scion 456-gas chromatograph equipped with Bruker SHS-40 Headspace Sampler coupled to a Bruker SQ mass spectrometer. The system was fitted with a Br-5 capillary column (30 m × 0.25 mm i. d. × 1.0 µm film thickness). The carrier gas was helium 5.0, at a constant flow rate of 1 mL min^−1^. Prior to sampling, headspace vials were incubated at 40 °C for 20 min in the automatic sampler with no agitation. Then, 1000 µL headspace samples were injected into the column. The transfer line and injector temperatures were maintained at 230 and 250 °C, respectively; a 20:1 split ratio was used. The oven initial temperature of 40 °C was held for 2 min, then increased to 280 °C at 10 °C/min, and held there for 3 min. The mass spectrometer operated in EI (70eV), source temperature: 180 °C; scanning rate: 1 scan s^−1^; mass spectra were recorded in full scan mode 50–400 m/z. Identification of the volatile compounds was based on mass spectrometric data obtained from the NIST (Version 2005) mass spectral library.

Method performance characteristics were estimated from calibration curves of mixtures of all authentic standards, which contained 0.01, 0.05, 0.10, 0.50, 1.00, 5.00, and 10.00 nL of analytes. Not all analytes were detected at each concentration level though; limit of quantitation values were chosen to be the lowest concentration point that was reproducibly detected. Intraday and interday relative standard deviations were calculated from at least 3 replicates from each point. For LOQ and equations, all data are presented in mmol (Supplementary Table S3).

### 4.7. Data Evaluation

For higher signal/noise ratio peak integration, extracted ion chromatograms (EIC, XIC) were integrated, as follows. First, all chromatographic peaks were selected manually, by examining the overlaid chromatograms. For each peak, the most abundant characteristic ions (1–5) from the cleanest spectrum (a sample showing a high peak) were collected manually. Using this list of characteristic ions, a targeted peak detection was run in mzMine 2.39 [51], followed by summation of the ion abundances belonging to the same metabolite, in R 4.0.2 [52]. Targeted peak detection parameters were: Intensity tolerance = 50.0%, Noise level = 9.0 × 10^4^, *m*/*z* tolerance = 0.5, Retention time tolerance = 0.1 (min). The data were converted to CDF format using Openchrom 1.4.0 [53]. All subsequent statistical tests were run in R. To test the statistical significance of the difference between the fungi with regard to metabolite abundances, abundance raw data were submitted to ANOVA models (*n* = 4 for each fungal strain). Dunnett post hoc tests were subsequently run on statistically significant compounds to obtain statistical differences for each fungus vs. control. To test the statistical difference between endophytes and soil fungi, the presence of individual compounds for each fungal strain was averaged to a single number and submitted to Fisher’s exact test.

## 5. Conclusions

The high relative surface agar film method was shown to be a suitable method for examination of VOC of endophytic fungi, growing on the medium prepared from their host plant’s tissues.

A large set of fungal genera were checked for VOC for the first time. The VOCs included not only the rather widespread alcohols, esters, and ketones, but also sulfur-containing volatiles that are likely decomposition products of the thioglucoside glucosinolates, abundant in horseradish extract. Not only was the usual allyl cyanide and allyl isothiocyanate detected in many instances, a significant increase in carbon disulfide and dimethyl sulfide also appeared, suggesting possible alternative decomposition or detoxification routes of the fungitoxic isothiocyanates.

The VOC produced from the host tissues can have various biological effects on the organisms in the environment of the fungi, as it includes antimicrobial compounds as well as compounds that can be utilized as a carbon source.

## Figures and Tables

**Table 1:** List of identified horseradish root endophytes and soil fungi used in the study.

Isolate	Species	Sequenced Gene(s)	Isolate	Species	Sequenced Gene
E1	*Fusarium oxysporum species complex*	ITS, eF1	S1	*Notophoma sp.*	ITS
E2	*Macrophomina phaseolina*	ITS, β-tubulin	S2	*Penicillium sp.*	ITS
E3	*Fusarium oxysporum species complex*	ITS, eF1	S3	*Curvularia sp.*	ITS
E4	*Setophoma terrestris*	ITS, α-actin	S4	*Curvularia sp.*	ITS
E5	*Paraphoma radicina*	ITS, α-actin	S5	*Curvularia sp.*	ITS
E6	*Paraphoma radicina*	ITS, α-actin	S6	*Aspergillus sp.*	α-actin
E7	*Oidiodendron cerealis*	ITS	S7	*Fusarium sp.*	ITS
E8	*Fusarium sp.*	ITS	S8	*Fusarium sp.*	ITS
E9	*Fusarium sp.*	ITS	S9	*Penicillium sp.*	ITS
E10	*Fusarium sp.*	ITS	S10	*Fusarium sp.*	ITS
E11	*Plectosphaerella sp.*	ITS	S11	*Fusarium sp.*	ITS
E12	*Plectosphaerella sp.*	ITS	S12	*Fusarium sp.*	ITS
E13	*Pyrenochaeta sp.*	ITS	S13	*Penicillium sp.*	ITS
E14	*Volutella sp.*	ITS	S14	*Penicillium sp.*	ITS
E15	*Phomopsis sp.*	ITS	S15	*Fusarium sp.*	ITS
E16	*Cadophora sp.*	ITS	S16	*Fusarium sp.*	ITS
E17	*Colletotrichum sp.*	ITS	S17	*Penicillium sp.*	ITS
			S18	*Penicillium sp.*	ITS
			S19	*Fusarium sp.*	ITS
			S20	*Penicillium sp.*	ITS
			S21	*Paraphoma sp.*	ITS
			S22	*Penicillium sp.*	ITS
			S23	*Penicillium sp.*	ITS
			S24	*Aspergillus sp.*	calmodulin
			S25	*Fusarium sp.*	ITS
			S26	*Penicillium sp.*	ITS

**Table 2:** Volatile organic compounds (VOCs) identified in the headspace of endophytes of horseradish or soil fungi growing on horseradish extract medium, detected by headspace GC-MS. Similarity score was obtained from a sample that contained the compound of interest with the best signal-to-noise ratio.

Compound	Chemical Class	Retention Time (min)	Most Abundant Peaks * (*m*/*z*)	Identification Method (Similarity Score)
methyl-1-propanol	alcohol	4.42	56, 74	NIST library (818)
methyl-1-butanol isomer 1	alcohol	6.49	55, 57, 70	NIST library (880)
methyl-1-butanol isomer 2	alcohol	6.55	55, 57, 70	NIST library (908)
styrene	aromatic	9.80	104, 78, 51	NIST library (855)
benzaldehyde	aromatic	11.17	51, 77, 106	NIST library (819)
methyl formate	ester	2.09	60	authentic standard
methyl acetate	ester	2.99	74	NIST library (926)
ethyl acetate	ester	4.20	61, 70, 88	authentic standard
ethyl propionate	ester	6.03	57	NIST library (762)
propyl acetate	ester	6.09	61, 73	NIST library (711)
methyl-1-butanol acetate	ester	9.32	72, 99	NIST library (703)
acetone	ketone	2.58	58	authentic standard
beta-phellandrene	monoterpene	12.41	77, 93, 136	NIST library (852)
allyl cyanide (ACN)	nitrile	5.02	52, 67	authentic standard
phenylpropionitrile (PCN)	nitrile	15.85	65, 91, 131	authentic standard
acetic acid	organic acid	3.58	60	NIST library (661)
dimethyl sulfide (Me_2_S)	organo-sulfur	2.88	62	authentic standard
carbon disulfide (CS_2_)	organo-sulfur	3.14	76	authentic standard
allyl isothiocyanate (AITC)	organo-sulfur	9.58	60, 72, 99	authentic standard
2-phenylethyl isothiocyanate (PEITC)	organo-sulfur	19.18	65, 91, 163	authentic standard

*: The most abundant *m/z* from the EI spectra that were used for automatic quantitation of the compounds in mzMine.

## Supplementary Materials

The following are available online at https://www.mdpi.com/2218-1989/10/11/451/s1, Material S1: Gene sequences of the fungal strains, Table S1: VOCs detected from the headspace of endophytic and soil fungi growing on horseradish root extract, Table S2: Peak areas of the identified compounds in the headspace of fungal isolates grown on horseradish extract, using different cultivation techniques, Table S3: Headspace GC-MS performance metrics, with the authentic standards used in the study.
