# Supplementary material for: Volatile Organic Compounds (VOCs) of Endophytic Fungi Growing on Extracts of the Host, Horseradish (Armoracia rusticana)
Source: Metabolites. 2020 Nov 8;10(11):451. doi: 10.3390/metabo10110451 (PMC7695154; doi:10.3390/metabo10110451)
Supplement: Supplementary file 1 [file metabolites-10-00451-s001.zip › Supplementary table 1.pdf]

Supplementary Table 1. VOCs detected from the headspace of endophytic and soil fungi growing on horseradish root extract.
Key: **+** = detected in a higher amount, compared to the control samples; **++** = detected in a considerably higher amount compared to the control samples (most of these are significantly higher compared to controls – ANOVA, followed by Dunnett test).
Abbreviations: AA, acetic acid; A, acetone; ACN, allyl cyanide; AITC, allyl isothiocyanate; B, benzaldehyde;BP, beta-phellandrene; CS2, carbon disulfide; Me2S, dimethyl sulfide; EA, ethyl acetate; EP, ethyl propionate; MA, methyl acetate; MF, methyl formate; MBA, methyl-1-butyl acetate; MB, methyl-1-butanol;MP, methyl-1-propanol; PA, propyl acetate; S, styrene.

**ID**	**Species**	**Compound**																
		**AA**	**A**	**ACN**	**AITC**	**B**	**BP**	**CS_2**	**Me_2S**	**EA**	**EP**	**MA**	**MF**	**MBA**	**MB**	**MP**	**PA**	**S**
E1	*Fusarium oxysporum species complex*	+	++	+				+		++	++	+	+		++	++	++	+
E2	*Macrophomina phaseolina*			+		++				+	+				++	++		+
E3	*Fusarium oxysporum species complex*	++	+	+	+			+		+	++		+	++	++	++		+
E4	*Setophoma terrestris*	++	+		+			+					+		+			
E5	*Paraphoma radicina*				+									++	+			
E6	*Paraphoma radicina*				+									+				
E7	*Oidiodendron cerealis*	++							++									
E8	*Fusarium sp.*	++	+	++		+		+		++	++	++	+		++	++	+	+
E9	*Fusarium sp.*			+						++	++	+			++	++	+	+
E10	*Fusarium sp.*			+														
E11	*Plectosphaerella sp.*		+	+	+			+						+	+			
E12	*Plectosphaerella sp.*		+	+	++	+		++						++	++	+		
E13	*Pyrenochaeta sp.*			+							+				++	++		+
E14	*Volutella ciliata*	+		+	+			+							+			
E15	*Phomopsis sp.*	++	++	+			+	+					+		+			+
E16	*Cadophora sp.*		+		+				+					+				
E17	*Colletotrichum sp.*		+	+	+			++						++	++			
S1	*Notophoma sp.*				++			+						++	+	+		
S2	*Penicillium sp.*	++		+											+			
S3	*Curvularia sp.*	+		+	+			+	+					++	++	++		
S4	*Curvularia sp.*	+	+	+									+					
S5	*Curvularia sp.*	+	+	++				+							++			
S6	*Aspergillus sp.*	+	+	+									+					
S7	*Fusarium sp.*			++				+							++	++		+
S8	*Fusarium sp.*	++	+	+				+					+					
S9	*Penicillium sp.*	+	+	+				+										
S10	*Fusarium sp.*	+	+	+				+		++	+	++	+		++	++	+	
S11	*Fusarium sp.*	++	+	+				+		++	++	++	+		++	++	++	++
S12	*Fusarium sp.*	++	+	+				+		++	++	++	+		++	++	++	
S13	*Penicillium sp.*		+	+	+	+		+					++		+			
S14	*Penicillium sp.*		+	+														
S15	*Fusarium sp.*		+					+		++	+	+	+		++	++		
S16	*Fusarium sp.*	++	+	+	+			++			+		++	++	++			+
S17	*Penicillium sp.*			+														
S18	*Penicillium sp.*	+	++	+	+			++					++	++	+			+
S19	*Fusarium sp.*			++		++		+		++	++	++			++	++	++	+
S20	*Penicillium sp.*	++		+				+							+			+
S21	*Paraphoma sp.*		+	+	++			+					+	++	+			+
S22	*Penicillium sp.*	++	+	+				+							+	+		
S23	*Penicillium sp.*	++	+	+											+			
S24	*Aspergillus sp.*	++	+	+	+			+						++	+			+
S25	*Fusarium sp.*		+	+				+		+	+	++			++	++		
S26	*Penicillium sp.*		+	+					+									
