# Supplementary material for: Volatile Organic Compounds (VOCs) of Endophytic Fungi Growing on Extracts of the Host, Horseradish (Armoracia rusticana)
Source: Metabolites. 2020 Nov 8;10(11):451. doi: 10.3390/metabo10110451 (PMC7695154; doi:10.3390/metabo10110451)
Supplement: Supplementary file 1 [file metabolites-10-00451-s001.zip › Supplementary table 2.pdf]

**Supplementary table 2.** Area under curve (AUC) of the identified compounds in the headspace of fungal isolates grown on horseradish extract, using different cultivation techniques.
Abbreviation: hs, high relative surface agar film (propsed technique); l, liquid; nf, agar.

| Strain | H1 | H1 | H1 | H1 | H1 | H1 |
|---|---|---|---|---|---|---|
| Medium format | hs | l | nf | hs | l | nf |
| Day | 3 | 3 | 3 | 4 | 4 | 4 |
| acetone | 4202865 | 552942 | 1530881 | 6570502 | 2738860 | 3275885 |
| ACN | 1885693 | 3148245 | 3008135 | 2191987 | 3872182 | 5468480 |
| AITC | 0 | 0 | 0 | 321872 | 0 | 394428 |
| $CS_2$ | 5150051 | 0 | 2238920 | 3438926 | 0 | 4124258 |
| ethyl acetate | 21735721 | 223811660 | 64286664 | 16317071 | 196439810 | 54646789 |
| ethyl propionate | 295745 | 0 | 385810 | 233220 | 0 | 0 |
| methyl acetate | 0 | 1878800 | 0 | 0 | 1742895 | 0 |
| methyl formate | 0 | 0 | 0 | 0 | 0 | 0 |
| methyl-1-butanol acetate | 0 | 0 | 0 | 0 | 0 | 0 |
| methyl-1-butanol isomer 1 | 11022823 | 10240929 | 13480199 | 9854887 | 14790513 | 11603569 |
| methyl-1-butanol isomer 2 | 4412099 | 8072548 | 5262103 | 3852259 | 9298005 | 5128776 |
| methyl-1-propanol | 14584496 | 16198003 | 20506366 | 14598790 | 17659924 | 21662212 |
| propyl acetate | 0 | 0 | 0 | 0 | 0 | 0 |
| styrene | 8582260 | 19198299 | 7759870 | 658365 | 1362931 | 695401 |

| Strain | H12 | H12 | H12 | H12 | H12 | H12 |
|---|---|---|---|---|---|---|
| Medium format | hs | l | nf | hs | l | nf |
| Day | 3 | 3 | 3 | 4 | 4 | 4 |
| acetone | 762373 | 1094639 | 1272257 | 157489 | 2648479 | 2476522 |
| ACN | 3639241 | 788222 | 2484930 | 5988333 | 226358 | 6495034 |
| AITC | 0 | 0 | 1966720 | 0 | 0 | 2947701 |
| $CS_2$ | 2255956 | 0 | 3947120 | 2323653 | 0 | 8613367 |
| ethyl acetate | 0 | 0 | 0 | 0 | 0 | 0 |
| ethyl propionate | 0 | 0 | 0 | 0 | 0 | 0 |
| methyl acetate | 0 | 0 | 0 | 0 | 0 | 0 |
| methyl formate | 0 | 0 | 0 | 0 | 0 | 0 |
| methyl-1-butanol acetate | 0 | 0 | 0 | 0 | 0 | 0 |
| methyl-1-butanol isomer 1 | 1363424 | 546807 | 1345376 | 1523985 | 805406 | 1165317 |
| methyl-1-butanol isomer 2 | 839223 | 582648 | 820072 | 390669 | 872264 | 610978 |
| methyl-1-propanol | 0 | 0 | 0 | 0 | 0 | 0 |
| propyl acetate | 0 | 0 | 0 | 0 | 0 | 0 |
| styrene | 0 | 0 | 0 | 0 | 0 | 0 |

| Strain | H6 | H6 | H6 | H6 | H6 | H6 |
|---|---|---|---|---|---|---|
| Medium format | hs | l | nf | hs | l | nf |
| Day | 3 | 3 | 3 | 4 | 4 | 4 |
| acetone | 880100 | 0 | 748808 | 1746364 | 1109550 | 1930111 |
| ACN | 0 | 0 | 0 | 0 | 0 | 0 |
| AITC | 3576559 | 0 | 0 | 1931962 | 0 | 0 |
| $CS_2$ | 0 | 0 | 0 | 0 | 0 | 0 |
| ethyl acetate | 0 | 0 | 0 | 0 | 0 | 0 |
| ethyl propionate | 0 | 0 | 0 | 0 | 0 | 0 |
| methyl acetate | 0 | 0 | 0 | 0 | 0 | 0 |
| methyl formate | 0 | 0 | 0 | 0 | 0 | 0 |
| methyl-1-butanol acetate | 0 | 0 | 0 | 0 | 0 | 0 |
| methyl-1-butanol isomer 1 | 0 | 0 | 0 | 0 | 0 | 0 |
| methyl-1-butanol isomer 2 | 0 | 0 | 0 | 0 | 0 | 0 |
| methyl-1-propanol | 0 | 0 | 0 | 0 | 0 | 0 |
| propyl acetate | 0 | 0 | 0 | 0 | 0 | 0 |
| styrene | 0 | 728744 | 0 | 0 | 0 | 0 |

| Strain | S16 | S16 | S16 | S16 | S16 | S16 |
|---|---|---|---|---|---|---|
| Medium format | hs | l | nf | hs | l | nf |
| Day | 3 | 3 | 3 | 4 | 4 | 4 |
| acetone | 0 | 1444965 | 1175321 | 369343 | 5686363 | 1420317 |
| ACN | 0 | 2351007 | 802703 | 0 | 3063334 | 5157357 |
| AITC | 0 | 306472 | 0 | 0 | 162357 | 0 |
| $CS_2$ | 3360262 | 1471845 | 1429624 | 1794660 | 2265532 | 4334149 |
| ethyl acetate | 12476638 | 730162014 | 133316303 | 0 | 709100742 | 113853998 |
| ethyl propionate | 0 | 294946 | 0 | 0 | 0 | 0 |
| methyl acetate | 0 | 4068663 | 1580298 | 0 | 3726227 | 1445773 |
| methyl formate | 202856 | 624760 | 0 | 0 | 397816 | 0 |
| methyl-1-butanol acetate | 0 | 0 | 0 | 0 | 0 | 0 |
| methyl-1-butanol isomer 1 | 422089 | 4102740 | 4961334 | 326919 | 4103062 | 4618100 |
| methyl-1-butanol isomer 2 | 0 | 2753780 | 3082155 | 0 | 3027924 | 1919232 |
| methyl-1-propanol | 1801655 | 12431953 | 9475080 | 508936 | 13970974 | 9503573 |
| propyl acetate | 0 | 2595488 | 752166 | 0 | 2434927 | 430642 |
| styrene | 0 | 0 | 0 | 0 | 0 | 0 |

| Strain | S2 | S2 | S2 | S2 | S2 | S2 |
|---|---|---|---|---|---|---|
| Medium format | hs | l | nf | hs | l | nf |
| Day | 3 | 3 | 3 | 4 | 4 | 4 |
| acetone | 0 | 0 | 940226 | 787342 | 1199554 | 1882295 |
| ACN | 913781 | 496443 | 0 | 946442 | 1178278 | 0 |
| AITC | 88068964 | 0 | 0 | 19371219 | 699326 | 279082 |
| $CS_2$ | 8617044 | 669101 | 0 | 5726052 | 1066883 | 0 |
| ethyl acetate | 0 | 0 | 0 | 0 | 0 | 0 |
| ethyl propionate | 0 | 0 | 0 | 0 | 0 | 0 |
| methyl acetate | 0 | 0 | 0 | 0 | 0 | 0 |
| methyl formate | 0 | 0 | 0 | 0 | 0 | 0 |
| methyl-1-butanol acetate | 1393839 | 0 | 0 | 0 | 0 | 0 |
| methyl-1-butanol isomer 1 | 1378131 | 2392323 | 0 | 1122189 | 1934524 | 0 |
| methyl-1-butanol isomer 2 | 945123 | 1339629 | 0 | 757582 | 1340476 | 0 |
| methyl-1-propanol | 721820 | 3083722 | 0 | 156953 | 2336930 | 0 |
| propyl acetate | 0 | 0 | 0 | 0 | 0 | 0 |
| styrene | 0 | 0 | 0 | 0 | 0 | 0 |

| Strain | S25 | S25 | S25 | S25 | S25 | S25 |
|---|---|---|---|---|---|---|
| Medium format | hs | l | nf | hs | l | nf |
| Day | 3 | 3 | 3 | 4 | 4 | 4 |
| acetone | 0 | 883408 | 0 | 1124436 | 3108065 | 1086028 |
| ACN | 1442541 | 1954617 | 2422201 | 4283734 | 2483826 | 2996431 |
| AITC | 332605 | 0 | 0 | 792812 | 0 | 714574 |
| $CS_2$ | 1522220 | 0 | 1616398 | 3288127 | 897272 | 2311512 |
| ethyl acetate | 0 | 0 | 0 | 0 | 0 | 0 |
| ethyl propionate | 0 | 0 | 0 | 0 | 0 | 0 |
| methyl acetate | 0 | 0 | 0 | 0 | 0 | 0 |
| methyl formate | 0 | 0 | 0 | 0 | 0 | 0 |
| methyl-1-butanol acetate | 0 | 0 | 0 | 0 | 0 | 0 |
| methyl-1-butanol isomer 1 | 0 | 0 | 552184 | 0 | 0 | 434650 |
| methyl-1-butanol isomer 2 | 0 | 0 | 0 | 0 | 0 | 0 |
| methyl-1-propanol | 0 | 0 | 0 | 0 | 0 | 0 |
| propyl acetate | 0 | 0 | 0 | 0 | 0 | 0 |
| styrene | 3617097 | 8131050 | 0 | 0 | 5264007 | 0 |

| Strain | S4 | S4 | S4 | S4 | S4 | S4 |
|---|---|---|---|---|---|---|
| Medium format | hs | l | nf | hs | l | nf |
| Day | 3 | 3 | 3 | 4 | 4 | 4 |
| acetone | 1230451 | 1130905 | 1017116 | 1218793 | 2119399 | 1762362 |
| ACN | 0 | 0 | 0 | 581047 | 0 | 0 |
| AITC | 0 | 0 | 0 | 247053 | 300496 | 0 |
| $CS_2$ | 0 | 0 | 0 | 0 | 0 | 0 |
| ethyl acetate | 0 | 0 | 0 | 0 | 0 | 0 |
| ethyl propionate | 0 | 0 | 0 | 0 | 0 | 0 |
| methyl acetate | 0 | 0 | 0 | 0 | 0 | 0 |
| methyl formate | 0 | 0 | 0 | 0 | 0 | 0 |
| methyl-1-butanol acetate | 0 | 0 | 0 | 0 | 0 | 0 |
| methyl-1-butanol isomer 1 | 663954 | 1917959 | 0 | 502362 | 1954341 | 0 |
| methyl-1-butanol isomer 2 | 0 | 1011494 | 0 | 0 | 994208 | 0 |
| methyl-1-propanol | 797758 | 3220912 | 0 | 716098 | 3150803 | 0 |
| propyl acetate | 0 | 0 | 0 | 0 | 0 | 0 |
| styrene | 0 | 0 | 0 | 0 | 0 | 0 |
