# Supplementary material for: Volatile Organic Compounds (VOCs) of Endophytic Fungi Growing on Extracts of the Host, Horseradish (Armoracia rusticana)
Source: Metabolites. 2020 Nov 8;10(11):451. doi: 10.3390/metabo10110451 (PMC7695154; doi:10.3390/metabo10110451)
Supplement: Supplementary file 1 [file metabolites-10-00451-s001.zip › Supplementary table 3.pdf]

Supplementary table 3. Headspace GC-MS performance metrics, with the authentic standards used in the study. Abbreviations: LOQ, limit of quantitation (lowest point of calibration curve); RSD, relative standard deviation. Analytes: ACN, allyl cyanide; AITC, allyl isothiocyanate; CS_2, carbon disulfide; Me_2S, dimethyl sulfide; PCN, phenylpropionitrile; PEITC, phenylethyl isothiocyanate. Intraday and interday RSD was calculated from samples 0.005 µL of each authentic standard (3.35E-05 – 8.29E-05 mmol, depending on analyte), diluted with MeCN.

Analyte	LOQ average, mmol	Average R^2	Intraday RSD	Interday RSD	Example equation
acetone	5.79E-07	0.9958	17.80%	15.76%	y = 7.74E+11 x -9.58E+5
ACN	1.09E-06	0.9846	13.29%	0.54%	y = 1.37E+12 x -5.90E+6
AITC	1.02E-06	0.9515	17.10%	12.78%	y = 2.35E+12 x -1.10E+7
CS_2	6.64E-06	0.9725	8.43%	2.76%	y = 5.30E+11 x -2.55E+6
ethyl acetate	1.82E-06	0.9880	10.74%	15.26%	y = 1.13E+12 x -3.33E+6
Me_2S	8.84E-06	0.9782	18.41%	40.22%	y = 4.19E+11 x -1.92E+6
methyl acetate	2.33E-06	0.9921	15.55%	32.85%	y = 4.22E+11 x -9.88E+5
methyl formate	2.95E-06	0.9933	11.58%	62.14%	y = 1.42E+11 x -5.87E+5
PCN	5.96E-07	0.9488	22.15%	10.04%	y = 8.83E+12 x -3.07E+7
PEITC	2.85E-07	0.9514	25.05%	9.04%	y = 1.24E+13 x -3.31E+7
