# Supplementary material for: Volatile Organic Compounds (VOCs) of Endophytic Fungi Growing on Extracts of the Host, Horseradish (Armoracia rusticana)
Source: Metabolites. 2020 Nov 8;10(11):451. doi: 10.3390/metabo10110451 (PMC7695154; doi:10.3390/metabo10110451)
Supplement: Supplementary file 1 [file metabolites-10-00451-s001.zip › Supplementery Material 1.pdf]

Strain: **E8**
Identified genus: ***Fusarium***
ITS sequence:
CCTACTGGGAATCGAGGATGACAGCGGAGGGACATTACCGAGTTTACAACTCCCAAACCCCTGTGAACATA
CCACTTGTTGCCTCGGCGGATCAGCCCGCTCCCGGTAAAACGGGACGGCCCGCCAGAGGACCCCTAAACTC
TGTTTCTATATGTAACTTCTGAGTAAAACCATAAATAAATCAAAACTTTCAACAACGGATCTCTTGGTTCTG
GCATCGATGAAGAACGCAGCAAAATGCGATAAGTAATGTGAATTGCAGAATTCAGTGAATCATCGAATCTT
TGAACGCACATTGCGCCCGCCAGTATTCTGGCGGGCATGCCTGTTCGAGCGTCATTTCAACCCTCAAGCACA
GCTTGGTGTTGGGACTCGCGTTAATTCGCGTTCCTCAAATTGATTGGCGGTCACGTCGAGCTTCCATAGCGT
AGTAGTAAAACCCTCGTTACTGGTAATCGTCGCGGCCACGCCGTTAAACCCCAACTTCTGAATGTTGACCTC
GGATCAGGTAGGAATACCCGCTGAACTTAAGCATATCAATAAGCGGGAGGAACGAAAGATTCATTATAATT
CTTGT
BLAST query:
>Fusarium oxysporum strain 4099 internal transcribed spacer 1, partial sequence; 5.8S ribosomal RNA gene and internal transcribed spacer 2, complete sequence; and large subunit ribosomal RNA gene, partial sequence
Sequence ID: MN817702.1 Length: 540
Range 1: 1 to 540 Score:981 bits(531), Expect:0.0, Identities:537/540(99%), Gaps:0/540(0%), Strand: Plus/Plus

Strain: **E9**
Identified genus: ***Fusarium***
ITS sequence:
GTCTTGGGGCCCCGGCTGGGACAGCGGAGGGACATTACCGAGTTTACAACTCCCAAACCCCTGTGAACATA
CCACTTGTTGCCTCGGCGGATCAGCCCGCTCCCGGTAAAACGGGACGGCCCGCCAGAGGACCCCTAAACTC
TGTTTCTATATGTAACTTCTGAGTAAAACCATAAATAAATCAAAACTTTCAACAACGGATCTCTTGGTTCTG
GCATCGATGAAGAACGCAGCAAAATGCGATAAGTAATGTGAATTGCAGAATTCAGTGAATCATCGAATCTT
TGAACGCACATTGCGCCCGCCAGTATTCTGGCGGGCATGCCTGTTCGAGCGTCATTTCAACCCTCAAGCACA
GCTTGGTGTTGGGACTCGCGTTAATTCGCGTTCCTCAAATTGATTGGCGGTCACGTCGAGCTTCCATAGCGT
AGTAGTAAAACCCTCGTTACTGGTAATCGTCGCGGCCACGCCGTTAAACCCCAACTTCTGAATGTTGACCTC
GGATCAGGTAGGAATACCCGCTGAACTTAAGCATATCAATAAGCGGAGGAAGATAAAACCCAAAAAAAA
G
BLAST query:
>Fusarium oxysporum isolate Fox64 18S ribosomal RNA gene, partial sequence; internal transcribed spacer 1, 5.8S ribosomal RNA gene, and internal transcribed spacer 2, complete sequence; and 28S ribosomal RNA gene, partial sequence
Sequence ID: KJ562370.1 Length: 555
Range 1: 14 to 555 Score:983 bits(532), Expect:0.0, Identities:539/542(99%), Gaps:2/542(0%), Strand: Plus/Plus

Strain: **E10**
Identified genus: ***Fusarium***
ITS sequence:
TACTAGGGCAATGCGTGGGGGAACAGCGGAGGGACATTACCGAGGTTTACAACTCCCAAACCCCTGTGAAC
ATACCACTTGTTGCCTCGGCGGATCAGCCCGCTCCCGGTAAAACGGGACGGCCCGCCAGAGGACCCCTAAA
CTCTGTTTCTATATGTAACTTCTGAGTAAAACCATAAATAAATCAAAACTTTCAACAACGGATCTCTTGGTT
CTGGCATCGATGAAGAACGCAGCAAAATGCGATAAGTAATGTGAATTGCAGAATTCAGTGAATCATCGAAT
CTTTGAACGCACATTGCGCCCGCCAGTATTCTGGCGGGCATGCCTGTTCGAGCGTCATTTCAACCCTCAAGC
ACAGCTTGGTGTTGGGACTCGCGTTAATTCGCGTTCCTCAAATTGATTGGCGGTCACGTCGAGCTTCCATAG
CGTAGTAGTAAAAACCCTCGTTACTGGTAATCGTCGCGGCCACGCCGTTAAACCCCAACTTCTGAATGTTGA
CCTCGGATCAGGTAGGAAATACCC
BLAST query:
>Fusarium oxysporum isolate P45_ITS_G03_010.ab1 internal transcribed spacer 1, partial sequence; 5.8S ribosomal RNA gene and internal transcribed spacer 2, complete sequence; and large subunit ribosomal RNA gene, partial sequence
Sequence ID: MT032691.1 Length: 534
Range 1: 6 to 514
Score:913 bits(494), Expect:0.0, Identities:506/511(99%), Gaps:3/511(0%), Strand: Plus/Plus

Strain: **E11**
Identified genus: ***Plectosphaerella***
ITS sequence:
ACAAAAACCAACTGGTTTGTGACAGCGGAGGGATCATTACTGAGGTACTACACTCTCTACCCTTTGTGAAC
TATTATACCTGTTGCTTCGGCGGCGCCCGCGAGGGTGCCCGCCGGTCTCATCAGAATCTCTGTTTCGAACC
CGACGATACTTCTGAGTGTTCTTAGCGAACTGTCAAAACTTTTAACAACGGATCTCTTGGCTCCAGCATCGA
TGAAGAACGCAGCGAAACGCGATATGTAATGTGAATTGCAGAATTCAGTGAATCATCGAATCTTTGAACGC
ACATGGCGCCTTCCAGTATCCTGGGAGGCATGCCTGTCCGAGCGTCGTTTCAACCCTCGAGCCCCCGTGGCC
CGGCG
BLAST query:
>Plectosphaerella cucumerina isolate I652_ITS1_F07_027.ab1 internal transcribed spacer 1, partial sequence; 5.8S ribosomal RNA gene and internal transcribed spacer 2, complete sequence; and large subunit ribosomal RNA gene, partial sequence
Sequence ID: MT032542.1 Length: 519
Range 1: 1 to 345
Score:627 bits(339), Expect:2e-175, Identities:344/346(99%), Gaps:2/346(0%), Strand: Plus/Plus

Strain: **E12**
Identified genus: ***Plectosphaerella***
ITS sequence:
AATGTYACGTTGGTGTACCAGCGGAGGTACATTACTGAGTACTACACTCTCTACCCTTTGTGAACTATTATACC
TGTTGCTTCGGCGGCGCCCGCGAGGGTGCCCGCCGGTCTCATCAGAATCTCTGTTTCGAACCCGACGATAC
TTCTGAGTGTTCTTAGCGAACTGTCAAAACTTTTAACAACGGATCTCTTGGCTCCAGCATCGATGAAGAACG
CAGCGAAACGCGATATGTAATGTGAATTGCAGAATTCAGTGAATCATCGAATCTTTGAACGCACATGGCGC
CTTCCAGTATCCTGGGAGGCATGCCTGTCCGAGCGTCGTTTCAACCCTCGAGCCCCCGTGGCCCGGCGTTGG
GGATCTGCCACGGCAGGCCCCTAAAACCAGTGGCGGACCCGAAGGCCCTCTCCTTTGCGCAGTAGCATCAG
CCTCGCATTGGGATCCCTCGGCGTCCTGCCTCTAAACCCCCCACAAGTCCGCTCCGGCGGCACCAAGGTTG
ACCTCGGATCAGGTAGGAATACCCGCTGAACTTAAGCATATCAWTAAGCGGAGGGAA
BLAST query:
>Plectosphaerella cucumerina strain NQ5GI3 small subunit ribosomal RNA gene, partial sequence; internal transcribed spacer 1, 5.8S ribosomal RNA gene, and internal transcribed spacer 2, complete sequence; and large subunit ribosomal RNA gene, partial sequence
Sequence ID: MK183802.1 Length: 597
Range 1: 45 to 595
Score:990 bits(536), Expect:0.0, Identities:546/551(99%), Gaps:1/551(0%), Strand: Plus/Plus

Strain: **E13**
Identified genus: ***Pyrenochaeta***
ITS sequence:
GGGGACAGTRCGTTAGTGACCTGCGGAGGTACATTATTGTATAACGGGGGCCGGCGAGGGATTGCGCACTT
CGGTGCGCCTTTCTTCCCCGCCCTGTCTGATACTACCCATGTCTTTTGCGTACCAATTGTTTCCTCGGTGGGCT
TGCCCGCCGGTTGGACACTATAAAACCTTTTGTAATTGCAGTCAGCGTCAGAAAAACATAATAATTACAACT
TTCAACAACGGATCTCTTGGTTCTGGCATCGATGAAGAACGCAGCGAAATGCGATAAGTAGTGTGAATTGC
AGAATTCAGTGAATCATCGAATCTTTGAACGCACATTGCGCCCCTTGGTATTCCATGGGGCATGCCTGTTCG
AGCGTCATTTGTACCCTCAAGCTCTGCTTGGTGTTGGGTGTTTGTCCCGCTTTGCGCGTGGACTCGCCTTAAA
GCAATTGGCAGCCGGCAATCTGGTTATAGAGCGCAGCACATTTTGCGCTTCTTGCCATGGATGTCGGCATCC
ATCAAAGTACATTTTTTTTGCTCTTGACCTCGGGATCAGGTAGGGATAACCCGCTGAAACTTAAGCATATCA
AATAAGCCRGAGGAA
BLAST query:
>Pyrenochaeta sp. strain Py004 small subunit ribosomal RNA gene, partial sequence; internal transcribed spacer 1, 5.8S ribosomal RNA gene, and internal transcribed spacer 2, complete sequence; and large subunit ribosomal RNA gene, partial sequence
Sequence ID: MN537136.1 Length: 608
Range 1: 34 to 607 Score:996 bits(539), Expect:0.0, Identities:568/580(98%), Gaps:9/580(1%), Strand: Plus/Plus

Strain: **E14**
Identified genus: ***Volutella***
ITS sequence:
TTAACRRWTYTCGTTGGTGTACCAGCGGAGGGTACATTACCGAGTTTTCAACTCCCAAACCCCTGTGAACAT
ACCAATCGTTGCCTCGGCGGGAGAGCCCCGGGCGTTGGCGAAAGCCTCGCACCGGTATCAGGCGCCCGCC
GGAGGACCCAAACAAACTCTTGAATTCTAAAGTATCTTCTGAGTAATATAAGTTAAATAAATTAAAACTTTC
AACAACGGATCTCTTGGTTCTGGCATCGATGAAGAACGCAGCGAAATGCGATAAGTAATGTGAATTGCAGA
ATTCAGTGAATCATCGAATCTTTGAACGCACATTGCGCCCGCCAGTATTCTGGCGGGCATGCCTGTTCGAGC
GTCATTTCAACCCTCAAGCCCAGCTTGGTGTTGGGGA
BLAST query:
>Volutella ciliata strain HSP113 small subunit ribosomal RNA gene, partial sequence; internal transcribed spacer 1 and 5.8S ribosomal RNA gene, complete sequence; and internal transcribed spacer 2, partial sequence
Sequence ID: MK433634.1 Length: 550
Range 1: 24 to 406 Score:691 bits(374), Expect:0.0, Identities:380/383(99%), Gaps:0/383(0%), Strand: Plus/Plus

Strain: **E15**
Identified genus: ***Phomopsis***
ITS sequence:
GKARARGGAACAKTTCGTTGGTGAMCAGCGGAGGTACATTGCTGGACGCGCTTCGGCGCACCCAGAAACC
CTTTGTGAACTTATACCTTACTGTTGCCTCGGCGCAGGCCGGCCTCACCGAGGCCCCTCGGAAACGAGGAG
CAGCCCGCCGGCGGCCAACCAAACTCTTGTTTCTTAGTGAATCTCTGAGTAAAAAAAACATAAATGAATCA
AAACTTTCAACAACGGATCTCTTGGTTCTGGCATCGATGAAGAACGCAGCGAAATGCGATAAGTAATGTGA
ATTGCAGAATTCAGTGAATCATCRAATCTTTGAACGCACATTGCGCCCTCTGGTATTCCGGAGGGMATGCCT
GTTCRAGCGTCATTTCAACCCTMAATGCATTGCTTAGSWGTGGAATTGWGTAAATGKTAAAAAAAATAATA
AAAAGAATAGAAAGAGGAAAATAAAAAAAAAAKTAAAATATTKAAASAAAAAAGAAKAGAAAAGAAS
AAAAAAMRGTAARGAAAGAAAAATAAMATCAAAAAWTAAAAAGAAAAGAATAAAATAAAAMYAGAA
ATAAAAAAACCAAATATGAAGGAAGTAAAAAAATARAGRAAAAAAAGAAAAAAAAAAWWASMTAKA
AGAAAGGATTGAATYSSAMMATGAGAAMGAGAGMGAGYSGCTAAATTTTMAAGAAGAAAGAGTGGATT
WTGWTWATKATTAAATAWAAATAAAAAASAAAAASAAAATAMMMAAAASAAAAAAAGAGAATTAASC
STAAAMAATCCCCCAAACMTCMAYCTAAAYTAAAATTTATAAATACCAAMAACAACCACMCCMAGGCCG
ACATAAAAAAATAGCCACCAAACCCAAWAACCCAAGAACASAAAAGAGSAAARGGCCAWTACCYCCCTT
AACAAACMCCMAACAATCAAAGCMACCAATAAAACTGTMGGYCAGAAAKAAAAAATGAAGAKGAAAA
AATAYAGAAATATAATTAAAATTGAAAAAAAAAAAAAAAAAGAAAGAKATGGMGCTSGGAGTCATATGGA
AKAWGSAYAATKGWAAMAAATATWTAAAAAAAATTTAAAASKAAAAAGMSAGGAATATTY
BLAST query:
>Phomopsis sp. isolate Phom1 small subunit ribosomal RNA gene, partial sequence; internal transcribed spacer 1, 5.8S ribosomal RNA gene, and internal transcribed spacer 2, complete sequence; and large subunit ribosomal RNA gene, partial sequence
Sequence ID: MN450640.1 Length: 571
Range 1: 19 to 395 Score:649 bits(351), Expect:0.0, Identities:368/378(97%), Gaps:4/378(1%), Strand: Plus/Plus

Strain: **E16**
Identified genus: ***Cadophora***
ITS sequence:
ARTKKGACAATTCAGTTRGTGACCTGCGGGAGGTACATTACTAGAGCAAAGGATAGACAGCGCCCGTGGAG
CTCGCTCCCGGGGCTACCCTACTCCGGTAGGGTTTAGAGTCGTCGAGCCTCTCGAAGAAGCTCGGTCCTGAA
CTCCACCCTTGAATAAACTACCTTTGTTGCTTTGGCGGGCCGCCTCGTGCCAGCGGCTTCGGCTGTTGAGTG
CCCGCCAGAGGACCACAACTCTTGTTTTTAGTGATGTCTGAGTACTATATAATAGTTAAAACTTTCAACAACG
GATCTCTTGGTTCTGGCATCGATGAAGAACGCAGCGAAATGCGATAAGTAATGTGAATTGCAGAATTCRGT
GAATCATCGAATCTTTGAACGCACATTGCGCCCTCTGGTATTCCGGGGGGCATGCCTGTTCGAGCGTCATTA
TAACCACTCAAGCTCTCGCTTGGTATTTGGGGGTTCGCGGWTTCGCGGYCCCTAAAAATCAATKGGRRGGT
GCCCTGTWCGGCTCTRCRSCGTAATAATAMATCCTTCGSSKTTTTGGATTCCGGGTAAGGTAATAAWTTWG
GCSAAAAAACCCYTTAATTGTTTTTTAAAAGGWTGGAACTTCGGGAAAWAAGGTAAAAAAATAARCCAA
GSTGAAAACTTTRAARSMATAATWAAWAAAAAMGGARAGAGGAAAAGARARRAGGAAAGAAGAKGTTA
AAAARAAAAAAAAAAAAAAGAAAAAAAAAAAAAAGAAGAAAAAGAAAARAAAGAAAAAAAAAAA
GAAAAAAAAAAAGAAAGRAAAGGAAAAGTAAAGRAGGAAAAAAAWAAAAGAAAAAAGRAAAAAAG
AWAAGAGAAAGAAAAAAAAAGAAAAARGKAAAARAAAAGAGAAACAGMASAAAASAAAAAAAGAAAG
GAAAAAGAGGAGAAAAAAAAGAAGAAAAAAAAAAAAAAAAAAAAAAAAAAAAGAGAAAARTTAWAA
WAAAAAAGAAAGAAAAAAAAAAAAAAAAAAAAAAAAAAAAAA
BLAST query:
>Cadophora orchidicola voucher JS242014 18S ribosomal RNA gene, partial sequence; internal transcribed spacer 1, 5.8S ribosomal RNA gene, and internal transcribed spacer 2, complete sequence; and 28S ribosomal RNA gene, partial sequence
Sequence ID: KY271867.1 Length: 630
Range 1: 23 to 528 Score:833 bits(451), Expect:0.0, Identities:493/514(96%), Gaps:9/514(1%), Strand: Plus/Plus

Strain: **E17**
Identified genus: ***Colletotrichum***
ITS sequence:
AGAARCARWYGCGTTGGTGACCAGCGGAGGGTACATTACTGAGTACTACACTCTCTACCCTTTGTGAACTAT
TATACCTGTTGCTTCGGCGGCGCCCGCGAGGGTGCCCGCCGGTCTCATCAGAATCTCTGTTTTCGAACCCGA
CGATACTTCTGAGTGTTCTTAGCGAACTGTCAAAACTTTTAACAACGGATCTCTTGGCTCCAGCATCGATGA
AGAACGCAGCGAAACGCGATATGTAATGTGAATTGCAGAATTCAGTGAATCATCGAATCTTTGAACGCACA
TGGCGCCTTCCAGTATCCTGGGAGGCATGCCTGTCCGAGCGTCGTTTCAACCCTCGAGCCCCCGTGGCCCGG
CGTTGGGGGATCTGCCCACGGCAGGCCCCTAAAACCAGTGGCGGACCCGAAGGGCCCTTCTCCTTTGCGCA
AGWAAAMAAYAARCCTACGCATTGAGGWTKCCRTCGGGGAGAACCATGCCCTCTWAAGACCGCRCCCAC
AAAGGGAWAAGTTACRAGRAGAAWMCCRAAGGKTTAGAYWCTWCMGRATCMAAGKTA
BLAST query:
>Colletotrichum pisi isolate OTU560 internal transcribed spacer 1, partial sequence; 5.8S ribosomal RNA gene and internal transcribed spacer 2, complete sequence; and 28S ribosomal RNA gene, partial sequence
Sequence ID: GU934514.1 Length: 563
Range 1: 12 to 425 Score:741 bits(401), Expect:0.0, Identities:414/419(99%), Gaps:5/419(1%), Strand: Plus/Plus

Strain: **S1**
Identified genus: ***Notophoma***
ITS sequence:
CCTATAGGCTTTCGTAGGTGACTGCGGAGGACATTACCTAGAGTTGCGGGCTTTGCCTGCCATCTCTTACCCA
TGTCTTTTGAGTACCTTCGTTTCCTCGGCGGGTCCGCCCGCCGATTGGACAAAACTTAAACCCTTTGTAATTG
AAATCAGCGTCTGAAAAAACTTAATAGTTACAACTTTCAACAACGGATCTCTTGGTTCTGGCATCGATGAA
GAACGCAGCGAAATGCGATAAGTAGTGTGAATTGCAGAATTCAGTGAATCATCGAATCTTTGAACGCACAT
TGCGCCCCTTGGTATTCCATGGGGCATGCCTGTTCGAGCGTCATTTGTACCTTCAAGCTTTGCTTGGTGTTGG
GTGTTTGTCTCGCCTCTGCGCGCAGACTCGCCTCAAAACGATTGGCAGCCGGCGTATTGATTTCGGAGCGCA
GTACATCTCGCGCTTTGTAGTCTCAACGACGACGTCGAAGAGAGTACTTTTTTCACTCTTGACCTCGGATCAG
GTAGGGATACCCGCTGAACTTAAGCATATCAATTAAGCGGAGAGAATCCCCTTTTTTTTTCAAAAAATAG
BLAST query:
>Nothophoma quercina isolate R49 small subunit ribosomal RNA gene, partial sequence; internal transcribed spacer 1, 5.8S ribosomal RNA gene, and internal transcribed spacer 2, complete sequence; and large subunit ribosomal RNA gene, partial sequence
Sequence ID: MT420644.1 Length: 542
Range 1: 3 to 540 Score:948 bits(513), Expect:0.0, Identities:532/540(99%), Gaps:6/540(1%), Strand: Plus/Plus

Strain: **S2**
Identified genus: ***Penicillium***
ITS sequence:
CMWWMCAGKGRGGYCCTCTGGGTCACCTCCACCCGYWTTATCGACCTTMTTGCTTCGGTGGTTCGMCTCT
CCGCCGGCGGGTGGCTATTCATGATGCTTTRATTACRACATCCCTTATTAATTCCCACCCCACCCCCCCCTAG
CACKATACTTTTTTAATTTCTTCCCCCCTCACTCAAAATTACCATAACAAACTCAAACACCTCCCCCTCCCCT
CCCTTCCCTKCCTTTCCCCCCCCCACTTATCCTAAAACATTAAACAACCACKCCCACACCAACCCCCCCKCCC
ATCCRCCCCCCCCCTCKKAMARRTAATTTTCTTAATMTTWATYTARCATAAARMAAASMCGATAAAAACA
CAAAATAATAAATTTAAAAATTSTTAATAAAAAAAAAGKACCCCCCCGCCGKTCACACCCTCCTAAKTAA
AATAACWCCCCTCACCCCTCCTYCCATMCTTTTCTTSATTTYTTCTCTCCCTCCTGCCCCGMSCAGACGCYCA
TTCCCAGGAWCTTACGAWTGAGGGCCTCKGGGTCACTCCACGCGTTTATGWACTGTTGCTCGGGGGCGCCC
GGYCGCGGGGGGCTCGCCCGGGCCCGCCGCCAAAACATGAAGCGCCGAGAMGCGTCTGGCAWTACTAAA
AGTAAACTTCACAGGTACCTGGTTCGCTCATCAWAACCGSAATGSYTARCAKGTGAATGCRAATCGKGAACT
CMGTCTTGAAGCCMTGATTCSSYATCGGGGGGKAGCGGRAAATTKSCTCTYCTSTKMGRGGGGWWCCWG
ACCATAAAGSCAGCSCWCCTAAATKTGAGWTCAACMTCTGTCRCGTTGTAKKTCACTTTYTTTYSTGWGTCY
YGTSTGKWSTGWCCGGACTTTMATCAAKGGGGGAATTCACTSTSTGTTTCATCTGTCGCR
BLAST query:
>Penicillium lanosum strain 2# 10-2 No.110411-1 18S ribosomal RNA gene, partial sequence; internal transcribed spacer 1, 5.8S ribosomal RNA gene, and internal transcribed spacer 2, complete sequence; and 28S ribosomal RNA gene, partial sequence
Sequence ID: JX536388.1 Length: 525
Range 1: 12 to 117 Score:73.4 bits(80), Expect:2e-08, Identities:86/107(80%), Gaps:12/107(11%), Strand: Plus/Plus

Strain: **S3**
Identified genus: ***Curvularia***
ITS sequence:
AAAACTGTTCGTAAGTGACCTMGCGGAGGGTACATTACACAATACAATATGAAGGCCCGTAACACGGCTGG
ATTATTTTATTACCCTTGTCTTTTGCGCACTTGTTGTTTCCTGGGCGGGTTTCGCCCGCCACCAGGACCACACC
ATAAACCTTTTTTATGCAGTTGCAATCAGCGTCAGTACAACAAATGTAAATCATTTACAACTTTCAACAACG
GATCTCTTGGTTCTGGCATCGATGAAGAACGCAGCGAAATGCGATACGTAGTGTGAATTGCAGAATTCAGT
GAATCATCGAATCTTTGAACGCACATTGCGCCCTTTGGTATTCCAAAGGGCATGCCTGTTCGAGCGTCATTT
GTACCCTCAAGCTTTGCTTGGTGTTGGGCGTTTTTTTGTCTTTGGTTATGCCAAAGACTCGCCTTAAAACGAT
TGGCAGCCGGCCTCCTGGTTTCGCAGCGCAGCACATTTTTGCGCTTGCAATCAGCAATAGAGGGCGGCACT
CCATCAAGACACTTTTTTTCACGTTTGACCTCGGATCAGGTAGGGATACCCGCTGAACTTAAGCATATCAAT
AAGCGGAGG
BLAST query:
>Curvularia inaequalis strain 4Lss1 internal transcribed spacer 1, partial sequence; 5.8S ribosomal RNA gene, complete sequence; and internal transcribed spacer 2, partial sequence
Sequence ID: KC897660.1 Length: 577
Range 1: 1 to 576 Score:1037 bits(561), Expect:0.0, Identities:572/577(99%), Gaps:2/577(0%), Strand: Plus/Plus

Strain: **S4**
Identified genus: ***Curvularia***
ITS sequence:
ATWTWACAGTTCGTTAGTGTAMCTGCGGAGGTACATTACACAATACAATATGAAGGCCCGTGACACGGCTG
GATTATTTTATTACCCTTGTCTTTTGCGCACTTGTTGTTTCCTGGGCGGKKTTCGCCCGCCACCAGGACCRCA
CCATAAACCTTTTTTATGCAGTTGCAATCAGCGTCAGTACAACAAATGTAAATCATTTACAACTTTCAACAA
CGGATCTCTTGGTTCTGGCATCGATGAAGAACGCAKCGAAATGCGATACGTASTGTGAATTGCAGAATTCAG
TGAATCATCSAATCTTTGAACGSACATTGCGCCCTTTGGTATTCCAAAGGGCATGCCTGTTCGAGCGKCATTT
GTACCCTCAAGCTTTGCTTGGTGTTGGGCGTTTTTTTGTCTTTGGTTATGCCAAAGACTCGCCTTAAAACGAT
TGGCAGCCGGCCTCCTGGTTTCGCAGCGCAGCACATTTTTGCGCTTGCAATCAGCAATAKAGGGCGGCACT
CCATCAAGACACTTTTTTTCACGTTTGACCTCGGATCAGGTAGGGATACCCGCTGAACTTAAGCATATCATTA
AGCGGAGGAA
BLAST query:
>Curvularia inaequalis 18S ribosomal RNA gene, partial sequence; internal transcribed spacer 1, 5.8S ribosomal RNA gene and internal transcribed spacer 2, complete sequence; and 28S ribosomal RNA gene, partial sequence
Sequence ID: AF313409.1 Length: 605
Range 1: 33 to 605 Score:992 bits(537), Expect:0.0, Identities:558/573(97%), Gaps:1/573(0%), Strand: Plus/Plus

Strain: **S5**
Identified genus: ***Curvularia***
ITS sequence:
AGCATTCGTAAGTGTACCTGCGGAGGTACATTACCGAKCGAGATTCTCTGATTCACCTCCCWCCCGTGTTTAT
TGTCCCTTGTTGCTTCSGCGCTTGYKGCTCCCGGGCGGGGGTTCGCCCGCGMCCCCGACCACCCRTCCGCCT
TTTTTACTTGTTGCTGCAGCGTCTTGACAWCTGAGTMAATCATTTACTATTTCAACAACGGATCTCTTGKYTC
TGGSWTCSAKGAWSAAYGAAGARMAMTGCGAWAYGYAATGYGAATTGYRAAWTTCAGAGAAYCATCRAAY
CTTTGAACKYWCATTGCGCCCTGTGSCATTCGRAAKGSCATGCCTGTTCSAGCGYCATTTGTACCCTGCAAGC
CYTTGCTTGGTGTTGGGCGTGTTTTTTGTCTKTCSGYTATGCCCAAAGGACTMSCCTTCCAACRAKTGGCAG
CCGGMCTCCTGGYGKCSCASCRCAGCACRTTTTTGYGCTTGCAATCATSAATAGASGGCGGCACTTCRTCAA
KACACTTTTTAYTTCWYGTYYTGACCTCRSCATCGAGRTCAGGGTATACCMTGCTGCAACTKTAAAGTCATAT
CAWATAMAGCTGGAASSGAAAGGAA
BLAST query:
>Curvularia inaequalis isolate F2-3-3 internal transcribed spacer 1, partial sequence; 5.8S ribosomal RNA gene and internal transcribed spacer 2, complete sequence; and 28S ribosomal RNA gene, partial sequence
Sequence ID: KX349453.1 Length: 559
Range 1: 59 to 558
Score:380 bits(421), Expect:7e-101, Identities:385/519(74%), Gaps:24/519(4%), Strand: Plus/Plus

Strain: **S6**
Identified genus: ***Aspergillus***
α-actin sequence:
GGGRRWCCTYSRRMWGTTTTTCGTAAGTCCAACCAAAATCCCCCACAAGATCCCTCACAAKGGCAACCCTT
CCCCTCCTCCKGGGAAGTCATTCCCACCAACCCTTGCGTCAGCCCCTCGGCCAAGCTGGGGTTGGTCGGAG
AATTTGCCACTTTGGACGTCGAGTGGAATTGACCATCAAAAATCCAAATACTGACCAAAATCCATAGCCTC
CATTGTCGGTCGTCCCCGTCACCAKGGGWAAATATCCCCTTGTTCTCTCACTGTTTCTCGGCCTTGACCTGGC
GTCGTTGCTCTATGCGCTGTTTAAACGCATCGCAAGCATTCCTTGCAAAATTGTCTTCCARAAAACTAACGC
AATGCTCCAGTATCAKGATKGGTATGGGCCAGAAGAACTCGWAAACTTTTKGTTCTGTYMCTMTTTAAATA
TCTMCTMTTTAAACYTTTYATAATTGCTTATATTCTATAAAATAKATAAATAAKCCAATCAAGTCAKMAAA
AAAAAMMAATAWATAATAATTTYTTTTTTTYTTGTTAKTTATTATACTTATTTTTATTTATTTMAWACATTMA
AACTWATTYMATTTTTTKATTTTTWTTTTWTTWATCWAAATTAATATACTCCATCAACCATMTTATYTAATT
ATTKATATATTTAKTTTWGTTTTTTAATCCTATATTTTTCCTTCRTTTSTTTTYTYTTKRTSTGTTSTCGTCTATYA
YTTCCTCATTCTAWTTATAACTCTTTCTTTCCTTCTTTTTTYTTTTTTTTAATTCACKATTKWTWTYARATCAR
AYMCATATTTTCAMACTAATATATTCTTTCATTCTRTTTTTCAKTTTCTTTYCTCATTCTCAKTGATTTTCCATC
TSMAATTSCTCTCTTTGCCTGSCTTCCCTTGTTTATCCTSCACTTCSTCSTKTGTTATTTCTTTTTTTCTCTCYKTC
TSTGTSTACACAATAGRTGATGTAARTRKCTTTATTTCACTCCTCCASAATCAWAACGTTCTCAAMATTAKTAT
TCAGAATCTTCACAKSTCGTSKT
BLAST query:
>Aspergillus udagawae strain CBS DTO_283-D3 actin gene, partial cds
Sequence ID: KY808536.1 Length: 705
Range 1: 493 to 539 Score:80.5 bits(43), Expect:2e-10, Identities:45/47(96%), Gaps:0/47(0%), Strand: Plus/Plus

Strain: **S7**
Identified genus: ***Fusarium***
ITS sequence:
GAACGACGGACTCGTGTTGTGACAGCGGAGGACATTACCGAGTTTACACTCCCAAACCCCTGTGAACATACC
ACTTGTTGCCTCGGCGGATCAGCCCGCTCCCGGTAAAACGGGACGGCCCGCCAGAGGACCCCTAAACTCTG
TTTCTATATGTAACTTCTGAGTAAAACCATAAATAAATCAAAACTTTCAACAACGGATCTCTTGGTTCTGGC
ATCGATGAAGAACGCAGCAAAATGCGATAAGTAATGTGAATTGCAGAATTCAGTGAATCATCGAATCTTTG
AACGCACATTGCGCCCGCCAGTATTCTGGCGGGCATGCCTGTTCGAGCGTCATTTCAACCCTCAAGCACAG
CTTGGTGTTGGGACTCGCGTTAATTCGCGTTCCTCAAATTGATTGGCGGTCACGTCGAGCTTCCATAGCGTA
GTAGTAAAACCCTCGTTACTGGTAATCGTCGCGGCCACGCCGTTAAACCCCAACTTCTGAATGTTGACCTCG
GATCAGGTAGGAATACCCGCTGAACTTAAGCATATCATAAGCGGAGGAATCCCCCCAATACTTCATTTATTT
TTT
BLAST query:
>Fusarium oxysporum isolate Fox64 18S ribosomal RNA gene, partial sequence; internal transcribed spacer 1, 5.8S ribosomal RNA gene, and internal transcribed spacer 2, complete sequence; and 28S ribosomal RNA gene, partial sequence
Sequence ID: KJ562370.1 Length: 555
Range 1: 13 to 555 Score:966 bits(523), Expect:0.0, Identities:538/544(99%), Gaps:6/544(1%), Strand: Plus/Plus

Strain: **S8**
Identified genus: ***Fusarium***
ITS sequence:
ATTCCCTACCTGGATCCAAGGGTCACATTCAGAAGTTGGGGTTTACGGCGTGGCCGCGACGATTACCAGTAA
CGAGGGTTTTACTACTACGCTATGGAAGCTCGACGTGACCGCCAATCAATTTGGGGAACGCGATTTGACTCG
CGAGTCCCAACACCAAGCTGGGCTTGAGGGTTGAAATGACGCTCGAACAGGCATGCCCGCCAGAATACTG
GCGGGCGCAATGTGCGTTCAAAGATTCATGATTCACTGAATTCTGCAATTCACATTACTTATCGCATTTTGCT
GCGTTCTTCATCGATGCCAGAACCAAGAGATCCGTTGTTGAAAGTTTTGATTTATTTATGGTTTTACTCAGAA
GTTACATATAGAAACAGAGTTTAGGGGTCCTCTGGCGGGCCGTCCCGGGTTACGG
BLAST query:
>Fusarium verticillioides isolate FM2 small subunit ribosomal RNA gene, partial sequence; internal transcribed spacer 1, 5.8S ribosomal RNA gene, and internal transcribed spacer 2, complete sequence; and large subunit ribosomal RNA gene, partial sequence
Sequence ID: MK790042.1 Length: 679
Range 1: 259 to 667 Score:717 bits(388), Expect:0.0, Identities:404/411(98%), Gaps:4/411(0%), Strand: Plus/Minus

Strain: **S9**
Identified genus: ***Penicillium***
ITS sequence:
GACRGTTCTKAAGGTGACCTGCGGAGGWCTTACCGAGCGAGRTTCCTCTGARKTCMACCYTCCCMMCCCG
KTGKTTTWTCGTAMCYTGTTGCTTCGGCGGGCCCGCCTCACGGCCGCCGGGGGGCTTCTGCCCTCTGGCCC
GCGCCCGCCGAAGACACCWTTGAMCGCTGWCTGAARATTGCAGTCTGAGCTATTAGCTAAATAAGTTAAA
ACTTTCAGCAACGGATCTCTKGGTTCCGGCATCRATGAAAAACGC
BLAST query:
>Penicillium restrictum strain GZU-BCEC393-3 18S ribosomal RNA gene, partial sequence; internal transcribed spacer 1, 5.8S ribosomal RNA gene, and internal transcribed spacer 2, complete sequence; and 28S ribosomal RNA gene, partial sequence
Sequence ID: GU565105.1 Length: 573
Range 1: 1 to 239 Score:311 bits(168), Expect:1e-80, Identities:215/244(88%), Gaps:7/244(2%), Strand: Plus/Plus

Strain: **S10**
Identified genus: ***Fusarium***
ITS sequence:
AACAAGTTCGTTGGTGACCAGCGGAGGGTACATTACCGAGTTTACAACTCCCAAACCCCTGTGAACATACCT
ATACGTTGCCTCGGCGGATCAGCCCGCGCCCTGTAAAAAGGGACGGCCCGCCCGAGGACCCTAAACTCTGT
TTTTAGTGGAACTTCTGAGTAAAACAAACAAATAAATCAAAACTTTCAACAACGGATCTCTTGGTTCTGGC
ATCGATGAAGAACGCAGCAAAATGCGATAAGTAATGTGAATTGCAGAATTCAGTGAATCATCGAATCTTTG
AACGCACATTGCGCCCGCCAGTATTCTGGCGGGCATGCCTGTTCGAGCGTCATTTCAACCCTCAAGCTCAGC
TTGGTGTTGGGACTCGCGGTAACCCGCGTTCCCCAAATCGATTGGCGGTCACGTCGAGCTTCCATAGCGTAG
TAATCATACACCTCGTTACTGGTAATCGTCGCGGCCACGCCGTAAAACCCCAACTTCTGAATGTTGACCTCG
GATCAGGTAGGAATACCCGCTGAACTTAAGCATATCATTAAGCGGGAGGAAA
BLAST query:
>Fusarium sp. isolate DSM100287_DF16_RLCS18 small subunit ribosomal RNA gene, partial sequence; internal transcribed spacer 1, 5.8S ribosomal RNA gene, and internal transcribed spacer 2, complete sequence; and large subunit ribosomal RNA gene, partial sequence
Sequence ID: MT453272.1 Length: 1136
Range 1: 30 to 585 Score:979 bits(530), Expect:0.0, Identities:549/557(99%), Gaps:5/557(0%), Strand: Plus/Plus

Strain: **S11**
Identified genus: ***Fusarium***
ITS sequence:
GRWAYSSGRGTTTWMCTCCCAAACCCCTGTGAACATACCACTTGTTGCCTTCGGCGGATCAGCCCGCTCCCG
GTAAAACGGGACGGCCCGCCAGAGGACCCCTAAACTCTGTTTCTATATGTAACTTCTGAGTAAAACCATAA
ATAAATCAAAACTTTCAACAACGGATCTCTTGGTTCTGGCATCGATGAAGAACGCAGCAAAATGCGATAA
GTAATGTGAATTGCAGAATTCAKTGAATCATCGAATCTTTGAACGCACATTGCGCCCGCCAGTATTCTGGCG
GGCATGCCTGTTCKAGCGTCATTTCAACCCTCAAGCACAGCTTTGTGTTGGGATTTKATTTAATTCTTGTTCC
TYATATTGATTGGATTTTTATTTTTATWAMMTAYAGTTAYASCAAAACTTTTTTTACTTTTATYTTTTTCTTTTY
TTCTTCTTTTAACTACTCCTRTWATWWTTTCTATTTTTTTTTCA
BLAST query:
>Fusarium oxysporum f. sp. lycopersici strain FWC81 18S ribosomal RNA gene, partial sequence; internal transcribed spacer 1, 5.8S ribosomal RNA gene, and internal transcribed spacer 2, complete sequence; and 28S ribosomal RNA gene, partial sequence
Sequence ID: KC478641.1 Length: 561
Range 1: 55 to 410 Score:599 bits(324), Expect:5e-167, Identities:344/356(97%), Gaps:0/356(0%), Strand: Plus/Plus

Strain: **S12**
Identified genus: ***Fusarium***
ITS sequence:
AWWTWACGTTCGTTGGTGTACCAGCGGAGGGTACATTACCGAGTTTACAACTCCCAAACCCCTGTGAACAT
ACCTATACGTTGCCTCGGCGGATCAGCCCGCGCCCTGTAAAAAGGGACGGCCCGCCCGAGGACCCTAAACT
CTGTTTTTAGTGGAACTTCTGAGTAAAACAAACAAATAAATCAAAACTTTCAACAACGGATCTCTTGGTTC
TGGCATCGATGAAGAACGCAGCAAAATGCGATAAGTAATGTGAATTGCAGAATTCAGTGAATCATCGAATC
TTTGAACGCACATTGCGCCCGCCAGTATTCTGGCGGGCATGCCTGTTCGAGCGTCATTTCAACCCTCAAGCT
CAGCTTGGTGTTGGGACTCGCGGTAACCCGCGTTCCCCAAATCGATTGGCGGTCACGTCGAGCTTCCATAGC
GTAGTAATCATACACCTCGTTACTGGTAATCGTCGCGGCCACGCCGTAAAACCCCAACTTCTGAATGTTGAC
CTCGRATCAGGTAGGAAATACCCGCTGAAACTTWAAGCATATCAATWAAGSSGGRAGGAA
BLAST query:
>Fusarium equiseti isolate Fequi20 18S ribosomal RNA gene, partial sequence; internal transcribed spacer 1, 5.8S ribosomal RNA gene, and internal transcribed spacer 2, complete sequence; and 28S ribosomal RNA gene, partial sequence
Sequence ID: KJ562376.1 Length: 558
Range 1: 14 to 558 Score:957 bits(518), Expect:0.0, Identities:540/551(98%), Gaps:6/551(1%), Strand: Plus/Plus

Strain: **S13**
Identified genus: ***Penicillium***
ITS sequence:
AAGGGWMATTACCGAGCGAGATTCTCTGATTCAACCTCCCACCCGTGTTTATTGTACCTTGTTGCTTCGGCG
GGCCCGCCTCACGGCCGCCGGGGGGCATCTGCCCCCGGGCCCGCGCCCGCCGAAGACACCTTGAACTCTGT
ATGAAAATTGCAGTCTGAGTCTAAATATAAATTATTTAAAACTTTCAACAACGGATCTCTTGGTTCCGGCAT
CGATGAAGAACGCAGCGAAATGCGATACGTAATGTGAATTGCAGAATTCAGTGAATCATCGAGTCTTTGAA
CGCACATTGCGCCCCCTGGTATTCCGGGGGGCATGCCTGTCCGAGCGTCATTGCTGCCCTCAAGCCCGGCTT
GTGTGTTGGGTCTCGTCCCCCTTCCCGGGGGGACGGGCCCGAAAGGCAGCGGCGGCACCGCGTCCGGTCCT
CGAGCGTATGGGGCTTTGTCACCCGCTCTGTAGGCCCGGCCGGCGCTTGCCGATCAACCAAAAACTTTT
BLAST query:
>Penicillium fellutanum strain CBS 118477 small subunit ribosomal RNA gene, partial sequence; internal transcribed spacer 1, 5.8S ribosomal RNA gene, and internal transcribed spacer 2, complete sequence; and large subunit ribosomal RNA gene, partial sequence
Sequence ID: MT309667.1 Length: 900
Range 1: 159 to 650
Score:891 bits(482), Expect:0.0, Identities:490/493(99%), Gaps:3/493(0%), Strand: Plus/Plus

Strain: **S14**
Identified genus: ***Penicillium***
ITS sequence:
GRGGCAGTACGCAAGTGTACCTGCGGAGGTACATTACCGAGTGAGGGCCCTCTGGGTCCAACCTCCCACCC
GTGTTTATCGTACCTTGTTGCTTCGGCGGGCCCGCCTCACGGCCGCCGGGGGGCTTCTGCCCTCTGGCCCGC
GCCCGCCGAAGACACCATTGAACGCTGTCTGAAGATTGCAGTCTGAGCAATTAGCTAAATAAGTTAAAACT
TTCAACAACGGATCTCTTGGTTCCGGCATCGATGAAGAACGCAGCGAAATGCGATACGTAATGTGAATTGC
AGAATTCAGTGAATCATCGAGTCTTTGAACGCACATTGCGCCCCCTGGTATTCCGGGGGGCATGCCTGTCCG
AGCGTCATTGCTGCCCTCAAGCACGGCTTGTGTGTTGGGCCTCCGTCCTCCTCCCGGGGGACGGGCCCGAA
AGGCAGCGGMGGCACCGCGTCCGGTCCTCGAGCGTATGGGGCTTCGTCACCCGCTCTTGTAGGCCCGGMC
GGCGCTTGMCRAMACATCAAATCTTTTTCCRGGWTGACCTCGGATCARGWAAGGGATACCCCGCTTGAA
CATTAAARCATATCMAAWAAAGCGAGARGGAAA
BLAST query:
>Penicillium philippinense strain CBS 623.72 small subunit ribosomal RNA gene, partial sequence; internal transcribed spacer 1, 5.8S ribosomal RNA gene, and internal transcribed spacer 2, complete sequence; and large subunit ribosomal RNA gene, partial sequence
Sequence ID: MH860600.1 Length: 833
Range 1: 103 to 680 Score:950 bits(514), Expect:0.0, Identities:562/588(96%), Gaps:11/588(1%), Strand: Plus/Plus

Strain: **S15**
Identified genus: ***Fusarium***
ITS sequence:
AAACATTCGTTGGTGACTAGCGGAGGGTACATTACCGAGTTTCACTCCCGRCCCCTGTGACTACCTAKACGTT
GCCTCGGGGGRTCASCCCGCGCCCCGTAMAAAGGGACGGCCCGCCCGAGGACCCCTAAACTCTGTTTTTAG
TGGAACTTCTGAGTAAAACAAACAAATAAATCAAAACTTTCAACAACGGATCTCTTGGTTCTGGCATCGAT
GAARAACGCAGCAAAATGCGATAAGTAATGTGAATTGCARAATTCAGTGAATCATCGAATCTTTGAACGCA
CATTGCGCCCGCCAGTATTCTGGCGGGCATGCCTGTTCGAGCGTCATTTCAACCCTCAAGCTCAGCTTGGTG
TTGGGACTCGCGGTAACCCGCGTTCCCCAAATCGATTGGCGGTCACGTCGAGCTTCCATAGCGTAGTAATCA
TACACCTCGTTACTGGTAATCGTCGCGGCCACGCCGTAAAACCCCAACTTCTGAATGTTGACCTCGGATCAG
GTAGGAATACCCGCTGAAACTTAAGCATATCATTAARGCGGGAGGAA
BLAST query:
>Fusarium sp. NRRL 45996 18S ribosomal RNA gene, partial sequence; internal transcribed spacer 1, 5.8S ribosomal RNA gene, and internal transcribed spacer 2, complete sequence; and 28S ribosomal RNA gene, partial sequence
Sequence ID: GQ505760.1 Length: 1130
Range 1: 16 to 558 Score:898 bits(486), Expect:0.0, Identities:525/546(96%), Gaps:7/546(1%), Strand: Plus/Plus

Strain: **S16**
Identified genus: ***Fusarium***
ITS sequence:
AACAGTTCGTTAGTGACTAGCGGAGGTCTTACCGAGYGTGCKTTCCCCGAYCTCCTGCGCCCACCCCTGTTCG
TTGCCCCGGGGGGYCTCCCCGCGCCCCGYAMAARGGGACGGCCCGCCCGAKGACCCCTAAACTCTGTTTTT
AGTGGAACTTCTGAGTAAAACAAACAAATAAATCAAAACTTTCAACWACGGATCTCTTGGTTCTGGCATC
GATGAARAACGCRGCAWAAKGCGATAAGTAATGTGAATTGCARAATTCAKTGAATCRKCGAATCTTTGAA
CGCACATTGCGCCCGCCAGTWTTCTGGCGGGCATGCCTGTTCGAGCGTCATTTCRACCCTCAAGCTCARCTT
GGTGTTGGGACTCGCGGTAACCCKCGTTCCCCRAATCGATTGSCGGTCACGTCGAGCTTCCATAGCGTAGTA
ATCATACACCTCGTTACTGGTAATCGTCGCGGCCACGCCGTAAAACCCCAACTTCTGAATGTTGACCTCGGA
TCAGGTAGGAATACCCGCTGAACTTAAGCATATCATTAAAGCGGAGGAA
BLAST query:
>Fusarium sp. strain RS-2 internal transcribed spacer 1, partial sequence; 5.8S ribosomal RNA gene and internal transcribed spacer 2, complete sequence; and large subunit ribosomal RNA gene, partial sequence
Sequence ID: MT150615.1 Length: 528
Range 1: 30 to 513 Score:771 bits(417), Expect:0.0, Identities:455/484(94%), Gaps:0/484(0%), Strand: Plus/Plus

Strain: **S17**
Identified genus: ***Penicillium***
ITS sequence:
TTKTTKACAATTCGCAGTGACCTGCGGTAGGTACATTACCGAGCGAGAATTCTCTGATTCAACCTCCCACCC
GTGTTTATTGTACCTTGTTGCTTCGGCGGGCCCGCCTCACGGCCGCCGGGGGGCATCTGCCCCCGGGCCCGC
GCCCGCCGAAGACACCTAGAACTCTGTATGAAAATTGCAGTCTGAGTCTAAATACAAATTATTTAAAACTTT
CAACAACGGATCTCTTGGTTCCGGCATCGATGAAGAACGCAGCGAAATGCGATACGTAATGTGAATTGCAG
AATTCAGTGAATCATCGAGTCTTTGAACGCACATTGCGCCCCCTGGTATTCCGGGGGGCATGCCTGTCCGAG
CGTCATTGCTGCCCTCAAGCCCGGCTTGTGTGTTGGGTCTCGTCCCCCTTCCCGGGGGGACGGGCCCGAAA
GGCAGCGGCGGCACCGCGTCCGGTCCTCGAGCGTATGGGGCTTTGTCACCCGCTCTGTAGGCCCGGCCGGC
GCTTGCCGATCAACCAAAACTTTTTTCCAGGTTGACCTCGGAWCAAGGTAGGGATACCCGCTRAACTTAAG
CATAATCAATAAAGCGGRAGGAAA
BLAST query:
>Penicillium sp. P10-15b internal transcribed spacer 1, partial sequence; 5.8S ribosomal RNA gene and internal transcribed spacer 2, complete sequence; and 28S ribosomal RNA gene, partial sequence
Sequence ID: KT354993.1 Length: 592
Range 1: 16 to 592 Score:1005 bits(544), Expect:0.0, Identities:568/580(98%), Gaps:4/580(0%), Strand: Plus/Plus

Strain: **S18**
Identified genus: ***Penicillium***
ITS sequence:
TAWTWAWACARTCGTAGTGACCTGCGGAGGTACATTACCGAGCGAGAATTCTCTGATTCAACCTCCCACCC
GTGTTTATTGTACCTTGTTGCTTCGGCGGGCCCGCCTCACGGCCGCCGGGGGGCATCTGCCCCCGGGCCCGC
GCCCGCCGAAGACACCTTGAACTCTGTATGAAAATTGCAGTCTGAGTCTAAATATAAATTATTTAAAACTTT
CAACAACGGATCTCTTGGTTCCGGCATCGATGAAGAACGCAGCGAAATGCGATACGTAATGTGAATTGCAG
AATTCAGTGAATCATCRAGTCTTTGAACGCACATTGCGCCCCCTGGTATTCCGGGGGGCATGCCTGTCCGAG
CGTCATTGCTGCCCTCAAGCCCGGCTTGTGTGTTGGGTCTCGWCCCMCTTCCCGGARAGGACRGGGMCCG
AAAS
BLAST query:
>Penicillium sp. P10-16c internal transcribed spacer 1, partial sequence; 5.8S ribosomal RNA gene and internal transcribed spacer 2, complete sequence; and 28S ribosomal RNA gene, partial sequence
Sequence ID: KT354994.1 Length: 593
Range 1: 14 to 431 Score:715 bits(387), Expect:0.0, Identities:408/420(97%), Gaps:4/420(0%), Strand: Plus/Plus

Strain: **S19**
Identified genus: ***Fusarium***
ITS sequence:
GRRWYWWKRRTTTYACTTRCAAACCCCTGTGAACATACCACTTGTTGCCTCGGCGGATCAGCCCGCTCCCG
GTAAAACGGGACGGCCCGCCAGAGGACCCCTAAACTCTGTTTCTATATGTAACTTCTGAGTAAAACCATAA
ATAAATCAAAACTTTCAACAACGGATCTCTTGGTTCTGGCATCGATGAAGAACGCAGCAAAATGCGATAA
GTAATGTGAATTGCAGAATTCAGTGAATCATCGAATCTTTGAACGCACATTGCGCCCGCCAGTATTCTGGCG
GGCATGCCTGTTCKAGCGTCATTTCAACCCTCAAGCACAKCTTGGTGTTGGGACTCKCGTTAATTCKCGTTC
CTCAAATTGAWTGGCGGTCACGTCKAGCTTCCATAGCGTAKTAGTAAAACCCTCKTTACTGGTAATCGTCKC
GGCCACRCCGTTAATCCCCAACTTCTGAATGTTGACCTCKGATCATGTAGGAATACCTGCTGAACTTATKCAT
ATCAATTAGCGGARGTAGTA
BLAST query:
>Fusarium oxysporum isolate Ginrarsnl_1 small subunit ribosomal RNA gene, partial sequence; internal transcribed spacer 1, 5.8S ribosomal RNA gene, and internal transcribed spacer 2, complete sequence; and large subunit ribosomal RNA gene, partial sequence
Sequence ID: MN055701.1 Length: 578
Range 1: 74 to 574 Score:845 bits(457), Expect:0.0, Identities:482/501(96%), Gaps:0/501(0%), Strand: Plus/Plus

Strain: **S20**
Identified genus: ***Penicillium***
ITS sequence:
ARGAGCAGTGCGCAGTGACCTGCGGAGGTACATTACCGAGCGAGAATTCTCTGATTCAACCTCCCACCCGTG
TTTATTGTACCTTGTTGCTTCGGCGGGCCCGCCTCACGGCCGCCGGGGGGCATCTGCCCCCGGGCCCGCGCC
CGCCGAAGACACCTTGAACTCTGTATGAAAATTGCAGTCTGAGTCTAAATATAAATTATTTAAAACTTTCAA
CAACGGATCTCTTGGTTCCGGCATCGATGAAGAACGCAGCGAAATGCGATACGTAATGTGAATTGCARAAT
TCAGTGAATCATCGAGTCTTTGAACGCACATTGCGCCCCCTGGTATTCCGGGRGGMATGCCTGTCCGAGCGT
CATTGCTGMCCTCAAGCCCGGCTTGTGTGTTGGGTCTCGTCCCCCTTCCACRGGAAARAACRGRMMCCCG
AAA
BLAST query:
>Penicillium canescens strain HSP207 internal transcribed spacer 1, partial sequence; 5.8S ribosomal RNA gene and internal transcribed spacer 2, complete sequence; and large subunit ribosomal RNA gene, partial sequence
Sequence ID: MK543250.1 Length: 562
Range 1: 1 to 395 Score:697 bits(377), Expect:0.0, Identities:387/395(98%), Gaps:1/395(0%), Strand: Plus/Plus

Strain: **S21**
Identified genus: ***Paraphoma***
ITS sequence:
TSSRRAAGGWMWTACCTTTCATGCAGAAGAGTTGAGATGGTTGAGTATCTCGCCTCTCAATTCTCGCTGTAT
TTTACCCTTGTTTTTCTCATACTATTATTTCCTCGGCAGGCTCGCCTGCCGGGTGAAACAACTTCAAACCTGT
TTAATTTTCAATCAGCGTCTGAACAAATTAATAATTACAACTTTCAACAACGGATCTCTTGGTTCTGGCATC
GATGAAGAACGCAGCGAAATGCGATAAGTAGTGTGAATTGCAGAATTCAGTGAATCATCGAATCTTTGAAC
GCACATTGCGCCCCTTGGTATTCCATGGGGCATGCCTGTTCGAGCGTCATTTGTACCTTCAAGCTCTGCTTGG
TGTTGGGTGTTTGTCCTTGCTCTAGTGGCGGGACTCGCCTCAAAGTAATTGGCAGCCAGTGTTTTGGTTTTG
AAGCGCAGCACAAGTCGCGATTCAAGTCTATACGCTAGTTTCCACAAGTCTTTTATCACTTTTGACCTCGGAT
CAGGGT
BLAST query:
>Paraphoma radicina genomic DNA sequence contains ITS1, 5.8S rRNA gene and ITS2, culture collection UTHSC:DI16-209
Sequence ID: LT796835.1 Length: 587
Range 1: 55 to 553 Score:915 bits(495), Expect:0.0, Identities:498/499(99%), Gaps:1/499(0%), Strand: Plus/Plus

Strain: **S22**
Identified genus: ***Penicillium***
ITS sequence:
AAACARTACGTAAGTGACCTGCGGAGGTACATTACCGAGCGAGAATTCTCTGATTCAACCTCCCACCCGTGT
TTATTGTACCTTGTTGCTTCGGYGGGYCCGCCTCACGGCCGTCGGGGGKGTCTGTCTCCGGTTCCKTGCCCCGS
CKAGACMCCTTGAACTCTGTWTGAAAATTGCWKGCTGAGTCTAAMTATRAMTTATTTAAWACTTTCAWC
MAMSGATCWCTTGGKTMCGGCTTSGATGAAGAACSCAKSRAAATGSSATACRWAAKGTGAATTGCAGAAT
WCAKKGAAWCATCRAGKCTTTGAACGCWCATTGCGCCCCCTGGTATTCCGGGGGGAATGCCTGTCCGAGC
GTCRTTGMTGCCCTCATWRCCGGCTTAKGYTTTGAGGACTCGAACCCCAAWCCAGAAAA
BLAST query:
>Penicillium sp. P10-16c internal transcribed spacer 1, partial sequence; 5.8S ribosomal RNA gene and internal transcribed spacer 2, complete sequence; and 28S ribosomal RNA gene, partial sequence
Sequence ID: KT354994.1 Length: 593
Range 1: 14 to 387 Score:435 bits(235), Expect:1e-117, Identities:315/374(84%), Gaps:3/374(0%), Strand: Plus/Plus

Strain: **S23**
Identified genus: ***Penicillium***
ITS sequence:
GRAAACARTGAGTAGTGACCTGCGGAGGTACATTACCGAGCGAGAATTCTCTGATTCAACCTCCCACCCGTG
TTTATTGTACCTTGTTGCTTCGGCGGCCCGCCTCACGGCCGCCGGGGGGCATCTGCCCCCGGGCCCGCGCC
CGCCGAAGACACCTTGAACTCTGTATGAAAATTGCAGTCTGAGTCTAAATATAAATTATTTAAAACTTTCAA
CAACGGATCTCTTGGTTCCGGCATCGATGAAGAACGCAGMGAAATGCGATACGTAATGTGAATTGCAGAAT
TCAGTGAATCATCGAGTCTTTGAACGCACATTGCGCCCCCTGGTATTCCGGGGGGCATGCCTGACCGAGCGT
CATTGCTGCCCTCGAGCCCGGCTTGAGAACAAAARGAATAAATSAAAAATTCCCGGGAGGAAAC
BLAST query:
>Penicillium sp. P10-16c internal transcribed spacer 1, partial sequence; 5.8S ribosomal RNA gene and internal transcribed spacer 2, complete sequence; and 28S ribosomal RNA gene, partial sequence
Sequence ID: KT354994.1 Length: 593
Range 1: 13 to 388 Score:656 bits(355), Expect:0.0, Identities:369/376(98%), Gaps:2/376(0%), Strand: Plus/Plus

Strain: **S24**
Identified genus: ***Aspergillus***
calmodulin sequence:
CAYSRSSRCYKKWAWWWGAAAWWTTTGTTTGTMCGGTTCGAAAACCTTGAAAGCCTCTCGGATTTCCTCC
TCGGAGTCGGTGTCCTTCATCTTTCTGGCCATCATTGTGAGGAACTCTGATATATTTTTTTGAGTCAGTGATC
TGCCCAATATCCTGTAAGCCTAGAGGTTGTATCGTGAGTATATACCTGGGAAATCAATGGTGCCGTTGTTGTC
GGCGTCAACCTCGTTGATCATGTCCTGGAGTTCAGACTCGGAGGGGTTCTGCCCGAGCGAGCGCATCACAG
TGCCGAGTTCCTTGGTGGTAATCTGGCCTAATCGCATTCACACATTATCTATACCAGGTTAATTGATGGTGCT
AAATATTATGGGATAATCGAGGCTGAGCGTATGCWAWAACTTTGCCGGATTGGGTGGGTTGCACYTTTTAT
CTCTTTACTTTTTCCWGTTCATAGCTGATTTTTGTTATTTWTTTTTWTTTTTTTTATM
BLAST query:
>Aspergillus pseudodeflectus partial CaM gene for calmodulin, isolate CCF 4965
Sequence ID: LT558760.1 Length: 752
Range 1: 140 to 539 Score:671 bits(363), Expect:0.0, Identities:389/402(97%), Gaps:3/402(0%), Strand: Plus/Minus

Strain: **S25**
Identified genus: ***Fusarium***
ITS sequence:
GAWYSRRGTWYYAMTTCCAAACCCCTTGTGACATACCTATAACGTTGCCTCGGCGGCGCCCGTCTCCCCGTA
AAACGGGAGCGGCCCGCCAGAGGAAAACCAAACTCTTGTTTTAGAAGTATCTTCTGAGTAACAAAAATAA
TCAATTAAAACTTTCAACAACGGATCTCTTGGTTCTGGCATCGATGAAGAACGCAGCGAAATGCGATAAGT
AATGTGAATTGCAGAATTCAGTGAATCATCRAATCTTTGAACGCACATTGCGCCCGCCAGTACTCTGGCGG
GCATGCCTGTTCGAGCGTCATTTCAACCCTCWAGCCCCATCGGTTTGGTTTTTTGTWTCCATTTTMTAATATT
AATTCCC
BLAST query:
>Fusarium sp. BDS-2017a strain CGMCC3.17370 18S ribosomal RNA gene, partial sequence; internal transcribed spacer 1, 5.8S ribosomal RNA gene, and internal transcribed spacer 2, complete sequence; and 28S ribosomal RNA gene, partial sequence
Sequence ID: KX790415.1 Length: 527
Range 1: 59 to 375 Score:562 bits(304), Expect:5e-156, Identities:313/318(98%), Gaps:2/318(0%), Strand: Plus/Plus

Strain: **S26**
Identified genus: ***Penicillium***
ITS sequence:
TGGWGCAGTCGCAGTGACCTGCGGGAGGtaCatTACCGAGTGAGGGCCCTCTGGGTCCAACCTCCCACCCGT
GTTTATCGTACCTTGTTGCTTCGGCGGGCCCGCCTCACGGCCGCCGGGGGGCTTCTGCCCTCTGGCCCGCGC
CCGCCGAAGACACCATTGAACGCTGTCTGAAGATTGCAGTCTGAGCAATTAGCTAAATAAGTTAAAACTTT
CAACAACGGATCTCTTGGTTCCGGCATCGATGAAGAACGCAGCGAAATGCGATACGTAATGTGAATTGCAG
AATTCAGTGAATCATCGAGTCTTTGAACGCACATTGCGCCCCCTGGTATTCCGGGGGGCATGCCTGTCCGAG
CGTCATTGCTGCCCTCAAGCACGGCTTGTGTGTTGGGCCTCCGTCCTCCTCCCGGGGGACGGGCCCGAAAG
GCAGCGGCGGCACCGCGTCCGGTCCTCGAGCGTATGGGGCTTCGTCACCCGCTCTTGTAGGCCCGGCCGGC
GCTTGCCGACACATCAATCTTTTTTCCAGGTTGACCTCGGATCAGGTAGGGATACCCGCT
BLAST query:
>Penicillium sp. isolate AM038-P9T4R 18S ribosomal RNA gene, partial sequence; internal transcribed spacer 1, 5.8S ribosomal RNA gene, and internal transcribed spacer 2, complete sequence; and 28S ribosomal RNA gene, partial sequence
Sequence ID: KT264414.1 Length: 581
Range 1: 10 to 557 Score:989 bits(535), Expect:0.0, Identities:544/548(99%), Gaps:1/548(0%), Strand: Plus/Plus
